# Glioblastoma: A Pathogenic Crosstalk between Tumor Cells and Pericytes

**DOI:** 10.1371/journal.pone.0101402

**Published:** 2014-07-17

**Authors:** Elisabetta M. Caspani, Philip H. Crossley, Carolina Redondo-Garcia, Salvador Martinez

**Affiliations:** 1 Laboratory of Experimental Embryology, Institute of Neuroscience, University Miguel Hernández-Spanish National Research Council, Alicante, Spain; 2 Centro de Investigación Biosanitaria en Red de Salud Mental and Instituto Murciano de Investigación Biosanitaria-Arrixaca, Murcia, Spain; University of Michigan School of Medicine, United States of America

## Abstract

Cancers likely originate in progenitor zones containing stem cells and perivascular stromal cells. Much evidence suggests stromal cells play a central role in tumor initiation and progression. Brain perivascular cells (pericytes) are contractile and function normally to regulate vessel tone and morphology, have stem cell properties, are interconvertible with macrophages and are involved in new vessel formation during angiogenesis. Nevertheless, how pericytes contribute to brain tumor infiltration is not known. In this study we have investigated the underlying mechanism by which the most lethal brain cancer, Glioblastoma Multiforme (GBM) interacts with pre-existing blood vessels (co-option) to promote tumor initiation and progression. Here, using mouse xenografts and laminin-coated silicone substrates, we show that GBM malignancy proceeds via specific and previously unknown interactions of tumor cells with brain pericytes. Two-photon and confocal live imaging revealed that GBM cells employ novel, Cdc42-dependent and actin-based cytoplasmic extensions, that we call flectopodia, to modify the normal contractile activity of pericytes. This results in the co-option of modified pre-existing blood vessels that support the expansion of the tumor margin. Furthermore, our data provide evidence for GBM cell/pericyte fusion-hybrids, some of which are located on abnormally constricted vessels ahead of the tumor and linked to tumor-promoting hypoxia. Remarkably, inhibiting Cdc42 function impairs vessel co-option and converts pericytes to a phagocytic/macrophage-like phenotype, thus favoring an innate immune response against the tumor. Our work, therefore, identifies for the first time a key GBM contact-dependent interaction that switches pericyte function from tumor-suppressor to tumor-promoter, indicating that GBM may harbor the seeds of its own destruction. These data support the development of therapeutic strategies directed against co-option (preventing incorporation and modification of pre-existing blood vessels), possibly in combination with anti-angiogenesis (blocking new vessel formation), which could lead to improved vascular targeting not only in Glioblastoma but also for other cancers.

## Introduction

Glioblastoma Multiforme (GBM) is a highly invasive brain cancer, with prominent vascular involvement, characterized by twisted blood vessel [1] and infiltration along external vessel walls [2], which makes it resistant to treatment. Evidence from a rat GBM model has shown that early tumor vasculature forms by co-option of pre-existing brain blood vessels and precedes new vessel formation (angiogenesis) [3]. Vessel co-option also occurs during metastasis of other tumors, as recently demonstrated for the spread of breast cancer into the brain [4]. Furthermore, co-option is also responsible for tumor recurrence and metastasis following anti-angiogenic therapies, both in GBM and in other types of cancer [5]-[8]. Therefore, vessel co-option is likely to be a principle cause of malignancy, which occurs during tumor initiation/progression, metastasis and re-initiation after treatment. However, in contrast to angiogenesis that is well understood, the cellular and molecular bases of vessel co-option in tumors are currently unknown. The normal brain microvasculature is made up of narrow tubes (capillaries), consisting of endothelial cells surrounded by contractile pericytes, which function normally to regulate vessel tone and morphology [9], [10]. Because pericytes are located on the abluminal wall of blood vessels, they are good candidates for a role in mediating vessel co-option by tumor cells. Brain pericytes are pluripotential cells with stem cell properties [11]-[13], similar if not identical to the mesenchymal stem cells that occupy an equivalent perivascular location in bone marrow. There is a growing realization that, in addition to their critical role in maintaining blood vessel integrity and controlling blood flow, pericytes are also key players in other aspects of brain homeostasis and disease. For example, evidence suggests that they are regulators of innate immunity and, depending on the context, can mediate not only pro-inflammatory functions associated with host defense [14], but also the anti-inflammatory response to malignant tumors such as human GBM, which includes the inhibition of T cell function and local immunosuppression [15]. Consistent with a role in normal cerebral immunity, purified brain pericytes have been shown to be interconvertible with macrophages [16] and to behave as macrophage-like cells in culture, by phagocytosing plastic beads [17] and by secreting inflammatory cytokines such as IL-1β, TNF-α and IL-6. Moreover, pericytes play an additional role in maintaining a proper function of the brain-immune interface, by controlling the migration of leukocytes in response to inflammatory mediators [18]. Given that immune cells contribute to tumor progression [19], pericytes could therefore provide a critical node for local control of both vessel co-option and immune system modulation. Within established tumors, blood vessels are often dysmorphic, with abnormal pericyte coverage and either atypical or absent endothelium [20]. Recent research, aimed to understand the possible function of pericytes in tumor progression, has emphasized their role in new vessel formation during angiogenesis [21]. In co-culture experiments, pericytes have been shown to modulate the angiogenic response of endothelial cells to glioma cells [22]. Furthermore, the recent discovery that GBM stem cells can trans-differentiate into tumor pericytes during the process of angiogenesis [23] further emphasizes the contribution of perivascular cells to tumor growth. While these findings underline the role of pericytes in the establishment of new vessels, very little is known about pericyte function in tumor infiltration. It is now recognized, for example, that perivascular tumor invasion occurs in some types of cancers [24]. Recent discoveries in breast and colon carcinomas have proposed that paracrine crosstalk between tumor and stromal cells is able to promote tumor growth and motility [25], [26]. Nevertheless, up to now no information exists about the cell biology of tumor cell/pericyte interaction, either in GBM or in cancer in general.

Here we use mouse xenografts in combination with live imaging techniques of brain explants and GBM-cell/pericyte co-cultures on laminin-coated deformable silicone substrates to investigate the fundamental cellular mechanisms by which GBM cells exploit the surrounding vascular niche to promote tumor survival and invasion.

## Results

**Figure 1:**
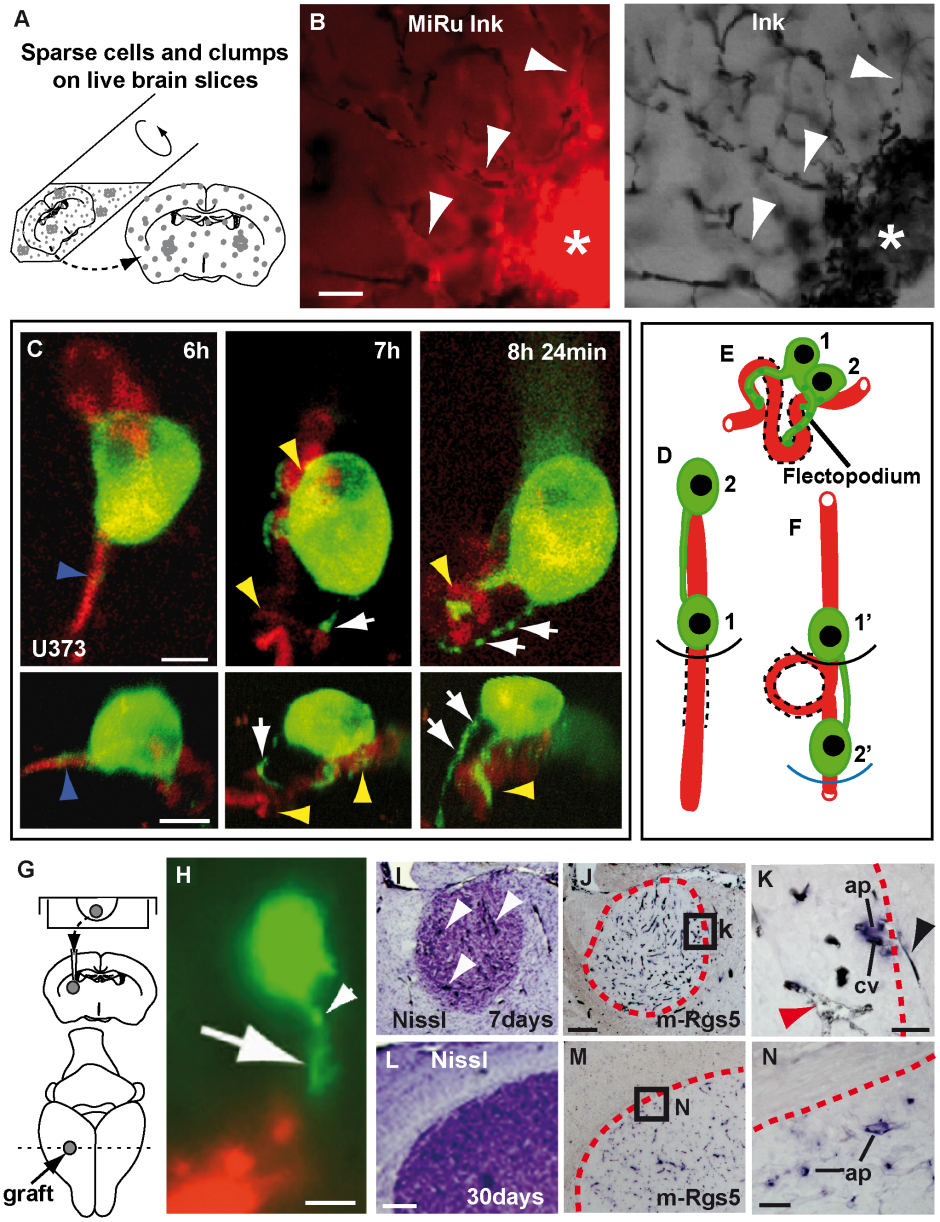
GBM cells co-opt and modify blood vessels *in-vivo*. **A**, Scheme showing seeding of human-GBM cells (grey spots) onto mouse brain slices. **B**, White arrowheads point to abnormal blood vessels (black-Ink), co-opted by fluorescence-labeled cells (MiRu^+^, red). Asterisks indicate agglomerated co-opted vessels in the body of the graft. **C**, Maximum projection (top) and 4D-reconstruction (bottom)-video frames (respectively) showing GFP-actin human GBM cells (green) re-arranging blood vessels. Frames have been selected to visualize flectopodia with GFP-actin-beads (green, white arrows) bending (yellow arrowheads) a previously straight vessel (DiI-red; blue arrowheads). **D**–**F**, A highly schematic cartoon of vessel structure before (**D**), during (**E**) and after (**F**) flectopodia-mediated co-option (based on Movie S1). 1 and 2, co-operating tumor cells (green), linked by cytoplasmic bridge; dashed-lines, recruited/modified vessel segment; black and blue arcs, which show the advance of GBM cells on the vessel, are analogous to the expanding tumor margin. **G**, Scheme of GBM-hanging drop xenografts. **H**, GFP-actin-U373 cell (green) in striatum of 2 day-xenograft, contacting host vessel (DiI-red) through a flectopodium (arrow) with moniliform-actin (white arrowhead). **I**, arrowheads point to Ink-filled, dilated vessels in 7 day-U87 graft. Activated perivascular cells (ap, Rgs5^+^, blue) are visible on co-opted, modified vessels (cv, black-Ink) at the expanding edge (red dashed-outlines) of both 7day- (**J**–**K**) and 1 month- (**M**–**N**) U87-xenografts. Note the difference in diameter between normal (black arrowhead) and **co**-opted (red arrowhead) vessels in **K**. Scale bars: 30 µm (**B**), 10 µm (**C**, **H**), 200 µm (**J**, **L**), 40 µm (**K**), 25 µm (**N**).

First, we challenged human GBM cells (U87) with mouse brain slices where blood vessels were pre-labeled with black ink (Figure 1A, B). This assay revealed a remarkable ability of GBM cells to pull pre-existing blood vessels into the graft. Vessel co-option happens quickly (within 15 hours) with conversion of normal capillaries into highly twisted structures, demonstrating a capacity for rapid vascular network acquisition, even in the absence of new vessel formation. Next, we used GFP-actin labeling and 2-photon live imaging to identify the intrinsic GBM cellular mechanisms involved in vessel-co-option (Figure S1; Figure 1C–F). We found that, 6 hours after seeding, tumor cells in contact with blood vessels can convert straight segments (up to ∼60–70 µm long) into hairpin bends (Figure 1C and Movie S1). Vessel re-organization is linked to the appearance of tumor cell protrusions, that we called ‘flectopodia’ (flectere, to bend, and podós, foot), characterized by an unusual, discontinuous (moniliform) organization of actin, with highly dynamic beads (0.6–3.0 µm in diameter), moving bidirectionally. Flectopodia are 7–30 µm long, present in 9% of GFP-actin^+^-cells on vessels (n = 266), with the ability to elongate for up to 20 µm or more, at a rate of 14–99 µm/hour, and to form a bent vessel segment in 30 minutes. A gap of approximately 10 µm separates the GFP-actin labeling from the vessel lumen, suggesting that flectopodia are in close contact with perivascular cells. Flectopodia-associated bending often involves a pair of GFP-actin labeled cells, cooperating via a cytoplasmic bridge. During the process, while the leading cell performs the re-arrangement, the lagging cell translocates the bent segment ahead of the first cell (Figure 1D–F; Movie S1). This suggests that flectopodia mediate the ongoing ratchet-like recruitment of host vessels at the co-option front of the tumor.

Next, to validate and extend our *ex-vivo* observations, we established a xenograft model that recapitulates human GBM in mice (Figure 1G) and used it to confirm the presence of flectopodia and vessel co-option *in situ*. High resolution-microscopy of sections from GFP-actin-labeled xenografts identified flectopodia-like extensions as early as 2 days (Figure 1H). *In situ* hybridization at 7 days for the activated mouse (m)-pericyte marker Rgs5, a gene controlling tumor vasculature remodeling [27], [28], showed the presence of m-Rgs5^+^-cells surrounding abnormally dilated vessels throughout the graft (Figure 1I–K). m-Rgs5^+^-pericytes are also detected in the infiltrating margin of 1-month xenografts (Figure 1L–N). Taken together these data strongly suggest that host brain perivascular cells are a target cell type for GBM vessel co-option and modification throughout tumor progression.

**Figure 2:**
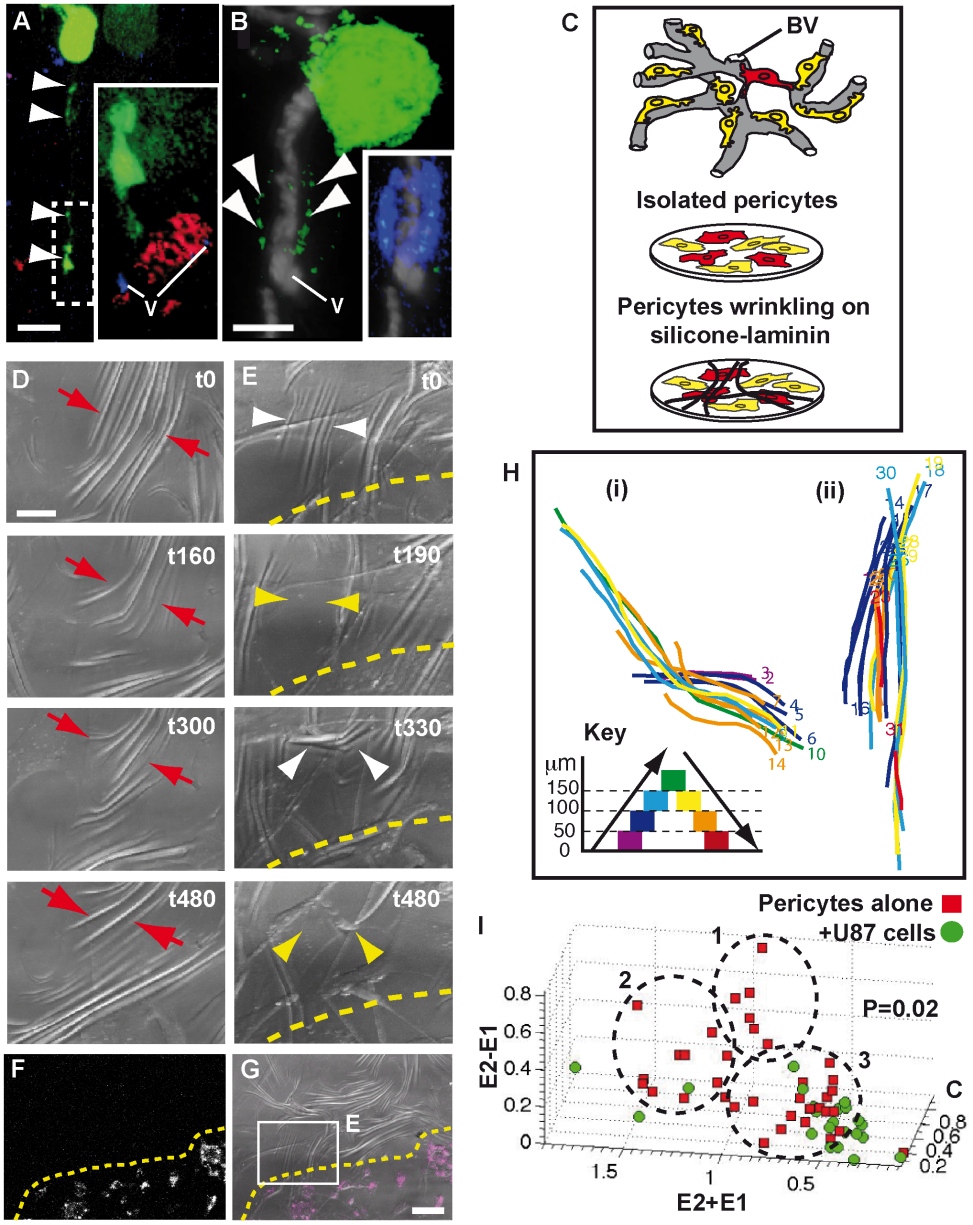
GBM cells target pericytes and modify their contractility. GFP-actin-GBM cells (green) contacting an NG2-DsRed^+^-pericyte (**A**, red iso-surface in magnified box) and a DLP (**B**, blue; confocal section) through flectopodia (arrowheads indicate moniliform actin). Note the presence of GFP-actin within the DLP (merged channels, inset in **B**). v, vessels (DiD-blue in **A**; Ink-filled-grey in **B**). **C**, Scheme showing pericyte (colored cells) *in-vivo* (top; BV, blood vessel), *in-vitro* (middle) and on silicone-substrate (bottom). Wrinkling is associated with high αSMA-expression (red-color). **D** and **E (**boxed area in **G)**, DIC-optic video-frames of the same field before (**D**) and after (**E**) GBM cell addition to pre-plated pericytes. Pericytes alone produce stable drifting wrinkles (red arrows) that are de-stabilized by GBM cells. White and yellow arrowheads indicate the appearance and disappearance of wrinkles, respectively. Dashed line marks the upper-limit of GBM cell population, transposed from **F** and **G**, which show the low magnification of FR Dextran-labeled GBM cells (white false-color in **F** and magenta in **G**), plated on cultured pericytes. Time in minutes. **H**, Traces of two wrinkles, produced before (i) and after (ii) U87-GBM cell-addition, revealing the spatial evolution and colored to indicate lengthening (violet to green) or shortening (green to red) for each time-frame (numbers). **I**, 3D-plot summarizing the wrinkling behavior of pericytes, either alone (red points, n = 40) or with U87-GBM cells (green points, n = 23). Note the lack of green points in clusters 1 and 2. E1, E2 and C: track-straightness of the ends (E) and center (C) of each wrinkle. Scale bars: 10 µm (**A**, **B**), 30 µm (**D**), 100 µm (**G**).

To confirm that GBM cells interact with pericytes, GFP-actin labeled U373 and U87 cells were co-cultured with brain slices pre-labeled *in situ* for pericytes, using either a DsRed-transgene reporter for the pericyte marker NG2 [9] or a fluorescent Dextran tracer (Methods). Our results show that tumor cells on vessels interact with Dextran (Dextran Labeled Pericytes, DLPs) or NG2-DsRed labeled perivascular cells (Figure 2A, B; Figure S2 A–G). Unexpectedly, we also found tumor-derived cytoplasm within the cortex of the target pericyte (Figure 2B and Figure S2 G), implying a role for cytoplasmic transfer in the co-option process (see also Movie S2).

Our identification of pericytes as a specific GBM cell target raised the possibility that the altered blood vessel morphology in tumors could be caused by deregulated pericyte contraction. To test this, we established an *in vitro* system using isolated mouse brain pericytes (Methods) cultured on deformable silicone substrates [29], with the novel coating of human-laminin to reproduce the blood vessel basal lamina that houses perivascular cells *in vivo* (Figure 2C). Pericytes *in vitro* express NG2 and display attributes consistent with stem (CD44; Vimentin; Nestin), contractile (α-smooth muscle actin, αSMA) and macrophage (CD68 and phagocytosis) potential (Figure S2 H). Two days after plating, cells generate compression forces (indicative of vasoconstriction activity [29]) visible, using Differential Interference Contrast (DIC) imaging, as wrinkles in the silicone sheet (Figure 2D). These are organized around local nodes of higher contractility (Movie S3), correlated with the expression of αSMA protein, a key determinant of pericyte contraction (Figure S2 I–K’). Remarkably, αSMA is also enriched in DLPs *in vivo*, suggesting that they may represent strategic nodes for the regulation of brain vessel tone (Figure S2 L–N’). Time-lapse confocal analysis showed that individual wrinkles change in position and length over time (between 20 and 200 µm) and cycle with a period of approximately 25/40 minutes (Movie S3; Figure S2 O–O’ and data not shown).

We then investigated if pericyte wrinkling-activity was affected by GBM cell addition. Although neither U373 nor U87 cells alone deform the substrate, in co-cultures they induce nearby pericytes to generate both new wrinkles and destabilize pre-existing ones (Figure 2E–G; Movie S4; Figure S2 P–P’). Quantification of the contractile activity with and without GBM cells, by tracking the behavior of identified wrinkles (Figure S2 O”, P”), revealed a difference in the way the wrinkles move in space and time (E2–E1, P = 0.02, where E1 and E2 are defined as track-straightness of each wrinkle end, Methods). Moreover and interestingly, including the track-straightness of the wrinkle center (C) and plotting all the wrinkle data in a 3D-scatter graph (Figure 2I), showed that GBM cells abolish the formation of wrinkles that drift laterally or drift laterally and pivot, typical of pericytes in nodes and inter-nodes (point clusters 1 and 2, respectively), leaving only the less organized activity characteristic of anti-nodes (point cluster 3; Movie S3). In conclusion, therefore, our data provide strong evidence that tumor cells can corrupt the intrinsic contractility of brain pericytes.

**Figure 3:**
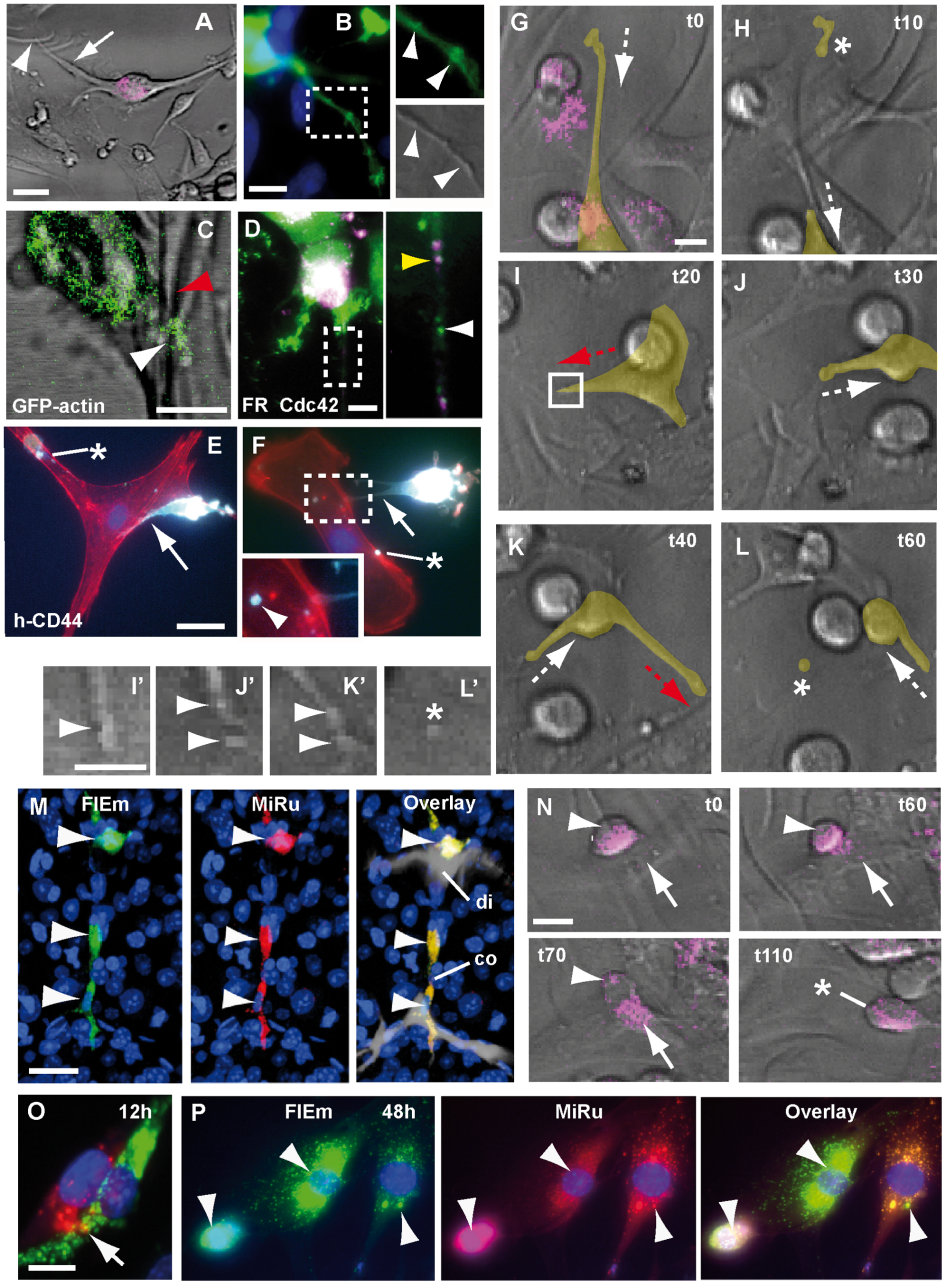
GBM cell/pericyte interaction involves flectopodia and cytoplasmic mixing. **A**, A flectopodia-like extension (arrow) from a FR labeled-GBM cell (magenta) contacts wrinkling pericytes (arrowhead) on silicone-laminin substrate (grey, DIC-optics). **B**, GFP-actin-beads in a presumptive flectopodia correlate with varicosities (arrowheads in insets). **C**, A beaded GFP-actin-extension (U87 cell, white arrowhead) induces altered wrinkling of pericytes (red arrowhead). **D**, Cdc42 protein (green, white arrowhead in the magnification) partially co-localizes with FR-dextran (FR, magenta, yellow arrowhead) as dots (0.5 µm in diameter) in the extension of a GBM cell. Fixed co-cultures show human CD44 protein in GBM cell flectopodia-like extensions (**E**–**F**, cyan, arrows) and in cytoplasmic particles (asterisks and arrowhead in magnification) in target pericytes (phalloidin, red). **G**–**L**, Time-lapse analysis of a U87 cell extending and retracting flectopodia (red and white dashed-arrows, respectively) and shedding terminal varicosities (asterisks, and magnifications in **H**, **L** and **L**’). The cell of interest was outlined and filled with a transparent yellow color using Photoshop. **M**, Double-labeled GDH cells (arrowheads) on constricted (co) and dilated (di) vessel segments (7 day-xenograft). **N**, Stepwise fusion-like process of a GBM cell (magenta, arrowheads) with a pericyte (arrows), resulting in a migratory cell-derivative (asterisk). Co-cultured GBM cells (MiRu^+^, red) and pericytes (FlEm^+^, green) show partial (**O**, arrow) or complete (**P**, white arrowheads) co-labeled cytoplasm, 12 or 48 hours after replating, respectively. Time in minutes. Scale bars: 30 µm (**A**, **E**), 10 µm (**B**, **C**, **G**, **I’**, **N**, **O**), 5 µm (**D**).

Next, we investigated the cellular mechanisms employed by GBM cells to induce pericyte dysfunction. Live imaging of U373 and U87 GBM cell/pericyte co-cultures on silicone-laminin substrates identified long extensions (maximum length 81±32 µm) (Figure 3A; Figure S2 P, P’), characterized by a discontinuous distribution of both cytoplasmic varicosities and GFP-actin (Figure 3B, C). Moreover, co-transfection assays *in vitro* showed that the small GTPase Cdc42, a principle regulator of cell polarity and actin cytoskeletal organization [30], is co-localized with the actin beads within cytoplasmic varicosities (Figure S3 A) and the native protein is visible within the GBM cell extensions on silicone substrate (Figure 3D). Importantly, analysis of human CD44, a cancer-associated cell surface adhesion molecule [31], revealed that the edges and tips of GBM cell elongations contact the target pericyte (Figure 3E, F). Remarkably, tumor cell projections predict the locations where the wrinkling pattern is changing (Figure S4 A-C”; Movie S5 and Figure 3C). Taken together, these converging lines of evidence strongly suggest that the cellular extensions seen on silicone substrates are similar, if not identical, to the flectopodia described above (Figure 1, 2). Flectopodia, therefore, are robust GBM cellular specializations that deregulate pericyte contractility.

Detailed analysis showed that flectopodia are characterized by alternating phases of extension and retraction (Figure 3G–L). Surprisingly, we found that, during retraction, cytoplasmic fragments (3–8 µm in diameter), corresponding to varicosities located originally at the advancing tip (Figure 3I’–K’) can be transferred into the target pericyte (Figure 3H, L and L’; Movie S6; Figure S4 D). These data are compatible with the GBM cell-cytoplasmic fragments positive for h-CD44 identified inside the contacted pericyte (Figure 3E, F) and corroborate cytoplasmic transfer observed in brain slices (Figure 2B; Figure S2 E, G). Moreover, this is supported by the mixing of GBM cell-derived Cherry-tagged Cdc42 and host DLP cytoplasms in xenografts (Figure S3 D–G). Taken together, our data indicate that co-option involves pericyte up-take of cytoplasmic micro-domains released by flectopodia.

Unexpectedly, our data suggest that GBM cells can use pericytes also as fusion-partners. First, MiniRuby-labeled (MiRu^+^) U373 grafts into unlabeled hosts generate clusters of strongly MiRu^+^-cells, which express mouse Rgs5 but lack human centromeric (h-cen) DNA (Figure S5 A, B). Furthermore, grafting Dextran MiRu^+^-U373 or U87 cells, into mouse brains or slices harboring DLPs, resulted in a range of differentially double-labeled derivatives (Figure S5 C–R). Among these, a novel cell type, that we called GDH (GBM cell/DLP Hybrid), is particularly striking due to its intense double labeling and location on highly constricted/dilated vessels, far beyond the tumor margin (Figure 3M; Figure S5 D, E, I). Surprisingly, GDH cells lack GBM cell-specific markers, h-CD44, h-Nestin, and h-cen DNA (Figure S5 K, M, O), but maintain high levels of αSMA, characteristic of the parental DLP (Figure S6 A–F). Critically, these curious double-labeled derivatives are strongly associated with both the presence of Nitrotyrosine (Figure S6 G–J) and hypoxia (Pimonidazole-staining, Figure S6 K–M), indicating that vessel hyper-contractility is linked to oxidative/nitrative stress [32]. GDH cells retain their strategic position even in advanced GBM-xenografts (Figure S6 N–U) and, interestingly, shed Cdc42^+^-particles in the lumen of dilated vessels in 7-day grafts, which are also found in sinusoidal vessels in 1-month-tumours (Figure S6 X, Y).

Remarkably, time-lapse confocal imaging on silicone substrates showed that the double-labeled cytoplasm, characteristic of GDH cells, could result from a fusion-like process that leads to the merger of a cell pair. This process, involving the interaction of a round GBM cell with a raised pericyte, occurs in three steps over 2–3 hours (Figure 3N and Movie S7). A preliminary cell/cell contact-phase (1-hour) is followed by tumor cell-cytoplasmic transfer and coalescence of the cell bodies (1-hour), with final translocation of the presumptive hybrid-derivative. When both GBM cells and pericytes were loaded with different colored Dextrans and combined on glass, we found cohorts of differentially double-labeled progenies (Figure 3O, P; Figure S7), some with aberrantly sized nuclei, indicative of abnormal ploidy. Time-course analysis showed that loss of h-CD44 and h-Nestin is already complete 48 h after cytoplasmic mixing (data not shown), supporting our cell-hybrid data in mouse xenografts (Figure S5). Thus, these results identify pericytes, for the first time, as a specific GBM cell-target for the production of fusion-like hybrids, with the potential to generate novel malignant cell variants, such as the hyper-contractile GDH cells, strategically located to maintain a hypoxic penumbra at the invasive edge of the tumor.

**Figure 4:**
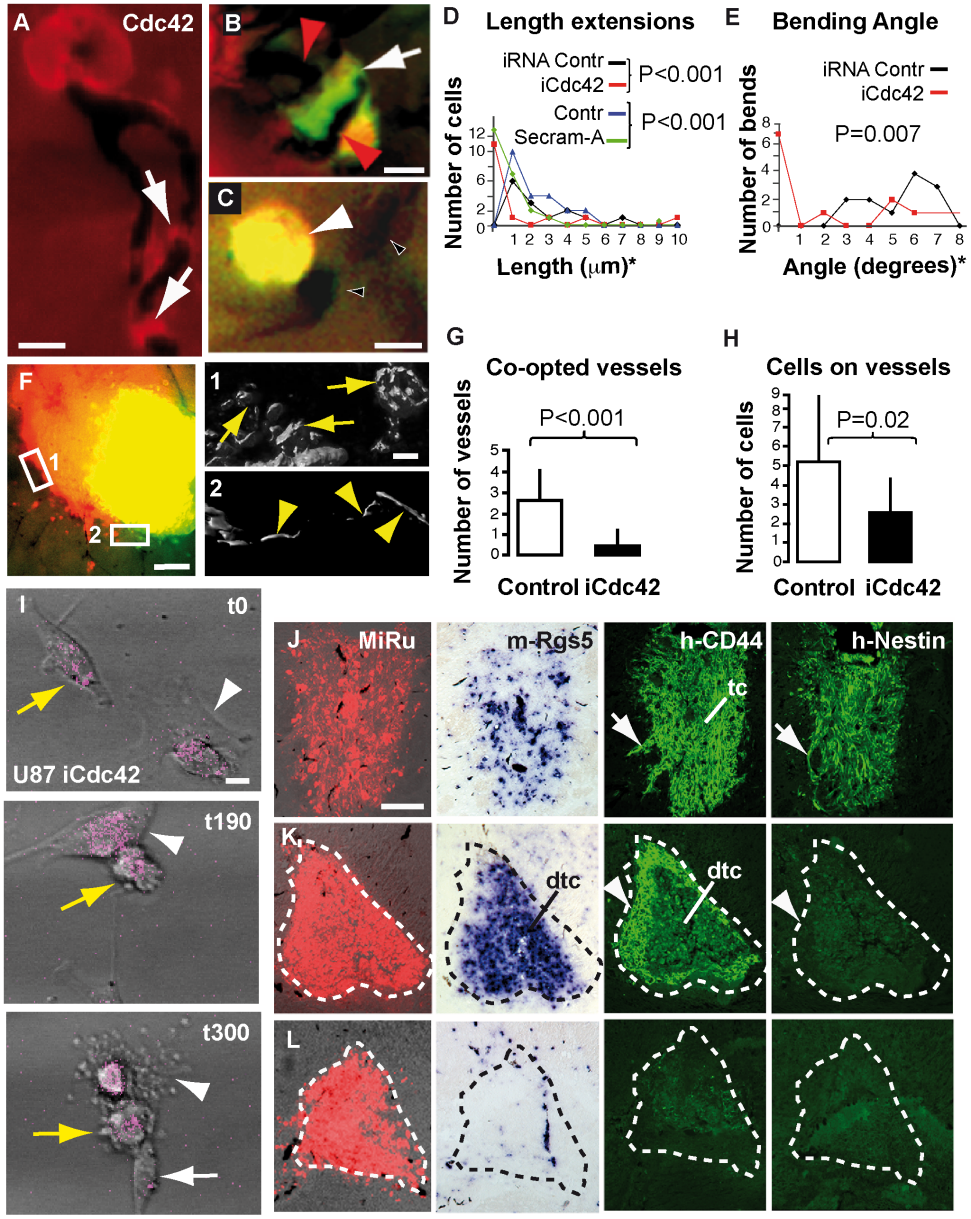
Cdc42-inhibition in GBM cells blocks flectopodia-mediated co-option and activates innate immunity. **A**, Cdc42 is present in U373 cell-flectopodia in brain slices (arrows). **B**, GFP-actin-labeled tumor cell (green; red color indicates transfection of the oligonucleotide siRNA control) co-opting a bent vessel (red arrowheads). **C**, A non-polarized, iCdc42-treated, GFP-actin^+^-U373 cell (yellow, arrowhead, indicates double labeling of green [GFP] and red [negative control for transfection]), on a straight vessel (Ink-filled, black-filled-white arrowheads). **D–E**, Graphs of the effects of iCdc42-treatment on the length of GBM cell extensions and the angle of vessel bending (asterisk, see Methods for length/angle-grouping). **D**, n = 15 (iRNA and iCdc42); controls, n = 22. **E**, n = 13 (controls and iCdc42). **F**, Two juxtaposed U373-grafts, with wild-type (MiRu^+^, red) or iCdc42 (FlEm^+^, green) cells. Magnifications display 3D rendering of Ink-filled co-opted convoluted (1, arrows) and non-co-opted straight (2, arrowheads) vessels, respectively. **G–H**, Quantitative analysis of graft/host margin interaction in short-term slice implants, incorporating both individual and juxtaposed grafts; n = 15 (all controls); n = 11 (iCdc42, **G**), n = 7 (iCdc42, **H**). **I**, Video-frames illustrating two macrophage-like cells (white arrowheads and arrow, respectively) pursuing and destroying a GBM cell (yellow arrows) (see also Movie S8). Time in minutes. U373-wild type (**J**, 7-day) or iCdc42 (**K**, 3-day and **L**, 7-day) xenografts analyzed for the indicated markers. Tc and dtc, core and degenerating tumor-core; white arrows and arrowheads show infiltrating and smooth margin, respectively; dashed outlines mark the original graft. Scale bars: 10 µm (**A**–**C**, **F**1**, I**), 100 µm (**F**, **J**).

Next, considering that flectopodia are actin-based extensions from highly polarized cells, we reasoned that inhibition of the actin GTPase Cdc42 might block vessel co-option. Immunohistochemistry on GBM cells seeded onto brain slices showed that Cdc42 is enriched in flectopodia (Figure 4A). Reducing Cdc42 in tumor cells, using either siRNA (iCdc42) or the specific Cdc42-inhibitor Secramine-A [33], results in shortened extensions to vessels and in reduced angle of vessel bending in brain slices (Figure 4B-E). Secramine-A also decreases the likelihood of a bend occurring where a tumor cell is attached to a blood vessel (Figure S8 A, B). To test Cdc42-function at the tumor/host margin, GBM cell-pellets, with and without inhibition for Cdc42, were grafted either separately or adjacent to each other into brain slices (Figure 4F). Importantly, while wild type-cells pull some vessels into the graft and use others for radial migration, iCdc42-treated cells show little affinity for blood vessel and no co-option (Figure 4F–H; Figure S8 C, C’). Co-opted vessels present abnormal constrictions, dilations and localized hairpin bends, while vessels adjacent to iCdc42-grafts maintain a straight morphology (Figure 4F1 and F2, respectively).

We then showed that CD44, a GBM marker with fusogenic properties [34], is enriched at vessel contact sites and cooperates with Cdc42 in vessel co-option/modification in brain slices. Our data demonstrated that knocking down CD44 (by shDNA) in combination with Cdc42 increases the inhibitory effect of iCdc42 alone on flectopodia-length and the angle of vessel bending, with a reduction in the number of glomeruloid-like structures by 70% (Figure S8 D–H). Additionally, iCdc42 shifts tumor cell-phenotype from ensheathing/re-arranging vessels, to a loosely associated state, a tendency amplified when CD44 is also reduced (Figure S8 I, J). Taken together, these data suggest that Cdc42 and CD44 act synergistically during flectopodia-induced vessel co-option/modification.

Subsequently, we tested the effect of iCdc42-GBM cells on pericyte behavior on silicone/laminin substrates. In addition to the initial pericyte activation induced by wild-type GBM cells (Figure S9 A–B”, E–F”, M), confocal live imaging showed a further pericyte-transformation into hyper-activated macrophage/dendritic-like cell phenotype, capable of killing and engulfing iCdc42-treated tumor cells, with concomitant overall reduction in wrinkling activity (Figure 4I; Movies S8, S9; Figure S9 C–D”, G–H”, I–L). In summary, this study uncovers a switching role for Cdc42 not only in flectopodia-mediated vessel co-option, but also in suppressing the activation of pericytes into cytotoxic, macrophage-like phenotypes.

We next investigated whether iCdc42-treatment could block vessel co-option and promote an immune response *in vivo*. Seven-day xenografts of wild type-GBM cells show high levels of h-CD44 and h-Nestin, with m-Rgs5^+^-cells around dilated, co-opted vessels (Figure 4J). In contrast, iCdc42 tumors appear to be compromised. Three days after grafting, implants present only a thin h-CD44^+^-shell and an intense m-Rgs5^+^-core, with no evidence for h-Nestin^+^-cells or vessel co-option (Figure 4K; Figure S9 P). By 7-days, the h-CD44^+^-shell and the m-Rgs5^+^-core are reduced or even absent (Figure 4L), with an accumulation of vimentin^+^-microglia at the implantation site, demonstrating an increased host phagocytic response (Figure S9 Q). Taken together, our data indicate that Cdc42 activity in GBM cells favors tumor-establishment over clearance.

Targeting the Cdc42/CD44/actin/pericyte/hypoxia axis (demonstrated above), to block vessel co-option in patients, depends critically on the underlying mechanism being conserved from mouse to human. Strikingly, we found that 88% (7/8) of an unbiased sample of 8 primary human GBM tumors show abnormally elevated levels of *CDC42* and *CD44*, restricted to perivascular locations on abnormal blood vessels at the boundary between tumor tissue and normal looking brain (Figure S10 A–C), a feature reproduced in an independent GBM biopsy (Figure S10 E). Interestingly, these two genes are part of a coordinately expressed group of genes (synexpression group), that may function together in tumorigenesis, including Hypoxia induced factor-1 alpha (*HIF1Α*), the actin binding protein Transgelin (*TAGLN*, SM22) and Platelet-derived growth factor receptor beta (*PDGFRΒ*), markers of abnormal tumor vessels [35] and pericytes [36], respectively (Figure S10 A–C). Notably, all the tumors (2/8) that recur 6 months after radio- and chemotherapies correlate to those cases where expression of these genes are greatest. Taken together, this validates the Cdc42/CD44/actin/pericyte/hypoxia axis as a desirable target for GBM therapy.

**Figure 5:**
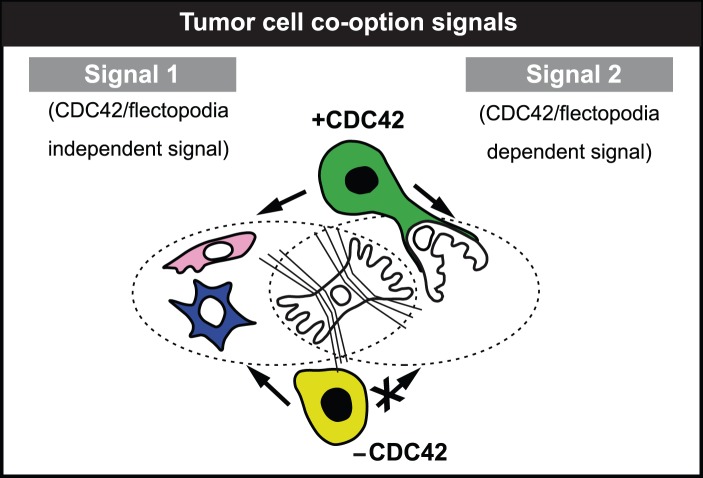
Tumor cell co-option signals. Two-signal model for GBM progression explains the effect of wild-type (green) and iCdc42 (light-green) GBM cells on inter-convertible, contractile pericytes (the white, wrinkling cell in the center). Blue, macrophage-like pericyte; pink and white, activated and co-opted pericytes, respectively.

Overall, our results suggest a 2-signal model for GBM progression that involves two distinct tumor cell-derived signals, which act on contractile brain pericytes (Figure 5 and Figure S11 A–D). The first signal (signal-1) causes pericyte activation and conversion to phagocytic, macrophage-like cells. In contrast, signal-2, which is flectopodia-dependent and requires active Cdc42 function, promotes vessel co-option by engaging contractile pericytes. The combination of these events generates fusion-like hybrids. In the presence of both signals, the tumor expands by continuous co-option and diversification, while, in the absence of signal-2, it is cleared by the unrestricted generation of cytotoxic cells, derived from the activation of contractile pericytes.

## Discussion

Recent advances in glioblastoma research have shown that GBM is propagated by perivascular stem-like cells [37] and that stemness can be transferred to ‘host’ cells during tumor progression [38]. Our discovery of a role for pericytes in vessel co-option provides a plausible explanation for these salient features of GBM. Recruitment of activated-pericytes may determine the perivascular location of tumor propagating cells, with transfer of stemness resulting from a fusion-like process and/or cytoplasmic inheritance. The requirement for ongoing co-option at the expanding tumor margin also suggests a reason for the failure of anti-angiogenic therapies, which target only new vessel formation [39].

In contrast to current cancer models that emphasize mutation and clonal expansion from a single-cell of origin [40], our work implies that glioblastoma is a dual cell of origin-disease, where tumor diversification is driven by GBM/perivascular cell coupling (Figure S11 E, F; Discussion S1).

A novel finding of this work is the discovery of flectopodia, as an important GBM cell specialization with the ability to alter vessel morphology. Flectopodia show a unique organization of actin in beads, which seems to reflect the ongoing trafficking of tumor cytoplasm into the cortex of the recipient pericyte. We found that vessel co-option and flectopodia formation and function are dependent on the activity of Cdc42 in tumor cells. Although the precise mechanism of Cdc42-action in this process remains to be determined, it likely relates to its well-established role as an evolutionary conserved organizer of the cortical actin cytoskeleton during cell polarization [30]. This suggests a number of possibilities. First, Cdc42 could act to polarize GBM cells towards the target and to initiate pericyte contact. Second, Cdc42 also controls the focal development of matrix-degrading structures called podosomes, found in normal cell types, such as macrophages and smooth muscle cells [41], and of functionally similar structures called invadopodia in several metastatic tumors [42] including glioma [43]. In spite of the dissimilarities in the actin organization in flectopodia and invadopodia (beads, described here, versus short filaments in bundles [44], respectively), it is intriguing to speculate that flectopodia represent the tissue-version of the *in-vitro* invadopodia. Alternatively, they might be GBM extensions highly specialized for the interaction with vessel pericytes. Finally, and perhaps more interesting, GBM cell-Cdc42 may act directly within the target pericyte to modify its behavior, as suggested by our demonstration that it is one of the proteins transferred (Figures S3 D-G and S7 B). In smooth muscle cells, physiological contraction in response to vasoconstrictor stimuli requires signaling through Rho-GTPase and Rho-kinase (ROCK), which act specifically by remodeling αSMA and myosin containing stress fibers. Smooth muscle cells also contain a second pathway that leads to actin polymerization and contraction, which is localized in the cortical cell cytoplasm and is mediated, through integrin activation by mechanical stress and contraction stimuli, by Cdc42 [45]. Since contractile pericytes behave similarly to smooth muscle cells and since overexpression of activated-Cdc42 is linked to hyper-contractility of the acto-myosin cortical cytoplasm [46], it is interesting to speculate, therefore, that local transfer of GBM-Cdc42 could cause inappropriate contraction of the cortical cytoskeleton, with consequent reorganization of blood vessel morphology. Our identification of Cdc42 as a molecular switch that drives flectopodia-mediated GBM cell/pericyte interaction reveals a new role for Cdc42 in addition to its previously proposed function in GBM cell migration [47], [48]. Interestingly, Cdc42 is implicated in regulating altered cell morphology downstream of TP53 [49] and PTEN [50], two tumor-suppressor genes mutated or inactivated in glioblastoma [51]. Hence, this suggests the intriguing possibility that Cdc42-mediated flectopodia formation results from inactivation of these two key regulatory genes.

Our findings also support pre-existing evidence indicating a direct molecular interaction of Cdc42 and CD44 in association with tumor cell-actin cytoskeleton [31], by demonstrating that they synergize in altering vessel architecture in brain slices. In particular, our data suggest that the adhesion protein CD44 could be involved in anchoring the cell to the vessel, facilitating the transfer/bending process.

The presence of Nitrotyrosine co-localized either within, or close to, fusion-like derivatives (GDH cells) suggests that these cells could represent a focal source of oxygen and nitrogen free radicals that might cause long lasting alterations in pericyte contraction. This is reminiscent of the Nitrotyrosine-containing pericytes that control the capillary ‘no-reflow’ phenomenon in ischemia reperfusion models of stroke [52]. The location of GDH cells on constricted and dilated blood vessels beyond the tumor margin indicates that they may contribute in creating the hypoxic penumbra, which surrounds the tumor core and which may promote tumor progression. It has also been suggested that reactive Nitrogen species and their derivatives, such as Nitrotyrosine, provide a “chemical barrier” that maintains both a tumor-specific immune response and supports the escape phase of cancer [53]. The presence of Nitrotyrosine associated with GDH cells, in our work, therefore implicates these strategically placed tumor cell/pericyte derivatives as part of such an immunological barrier.

Our work provides additional support for the idea that brain pericyte are part of the innate immune system and can behave as macrophage-like cells which possess phagocytic activity [17]. We have shown, here, that isolated brain pericytes *in vitro* express the macrophage [54] and general phagocytic [55] marker CD68 and are able to engulf fluorescent beads. Furthermore, the cell morphologies of our pericytes co-cultured with GBM cells on laminin-coated silicone substrates present a similarity to M1 and M2 polarized macrophage phenotypes described by others [56]. Specifically, our round and/or spindle-shaped pericytes, induced by wild-type tumor cells, resemble the anti-inflammatory M2, Tumor Associated Macrophages (TAMs) involved in tumor promotion. In contrast, ‘dendritic-like’ morphologies found in our pericyte co-cultures with iCdc42-GBM cells, which are able to pursue and phagocytose them, are more similar to pro-inflammatory, tumoricidal M1 macrophages. Thus, pericytes are highly plastic cells that can respond to GBM-mediated signals by changing their contractility, detaching from the substrate and acquiring a pro- or anti-tumor activity. Although it remains to be determined how our proposed two signal-model (Figure 5) fits with the well characterized signaling systems involved in tumor immunology, we speculate that it could involve inflammatory cytokine networks and purinergic signaling (Figure S11 G, H).

In conclusion, our findings reveal that brain pericytes not only provide the *materia prima* for GBM progression, through blood vessel co-option and immune suppression, but remarkably may also represent its ‘Achilles’ heel’. Taken together, Cdc42-inhibitors (possibly in combination with anti-angiogenic drugs) may prove therapeutically beneficial not only against primary and recurrent glioblastoma, but also other tumors that co-opt blood vessels during brain metastasis [57].

## Materials and Methods

### Human tumor cells and in vivo labeling

Human glioblastoma cell lines U87-MG and U373-MG were purchased from American Type Culture Collection (ATCC) and European Cell Culture Collection (ECACC), respectively, and grown in α-MEM medium with 10% fetal bovine-serum (Invitrogen). Cells for imaging were transfected with: GFP-actin plasmid (pEGFP-actin, Clontech), encoding a fusion of EGFP and human cytoplasmic beta actin; EGFP-plasmid alone (pCAGGS-GFP); Cherry-Cdc42 plasmid, encoding an N-terminal fusion of mCherry and human Cdc42 (GeneCopoeia); GFP-RR (for GFP targeting to the outer mitochondrial membrane, kind gift from Dr. Nica Borgese, CNR, University of Milano, Italy) and GFP-beta3 integrin (kind gift from Dr. Victor Small, Institute of Molecular Biotechnology, Vienna, Austria). For chemical transfection, we used Superfect reagent (Qiagen) according to manufacturer’s instructions. Dextran-labeled U87 and U373 cell pellets (biotinylated mini-Ruby, MiRu, Fluoro-Emerald, FlEm, or Alexa-Fluor-647: D3312, D7178 and D22914, respectively, Invitrogen/Molecular Probes) were prepared in 20 µl hanging drops [58], containing 45,000 cells, 17.5 µl growth medium and 2.5 µl of Dextran stock (0.5 mg in 40 µl H_2_O). Cultures were incubated for 48 hours and cell pellets were washed prior to use, either as grafts into mouse brain slices or as implants into mouse brains. In some experiments, single cell suspensions of Dextran-labeled cells were generated by trypsinization and trituration of labeled cell pellets. GBM cells for 2-day mouse xenografts were transfected with h-Cherry-Cdc42 and used to prepare hanging drops. In some cases GFP-actin transfected cells were co-labeled with CMTMR (5-6-4-Chloromethyl-Benzoyl-Amino-Tetramethylrhodamine).

#### Pericyte isolation and in vitro co-cultures

Mouse brain pericytes for co-culture experiments were isolated according to the method of Oishi et al. [59]. Pericytes and GBM cells were labeled separately with different color Dextrans, as hanging drops. Prior to co-culture, cell aggregates were dispersed to a single cell suspension with trypsin, mixed at a ratio of 1∶1 with GBM cells and plated on glass coverslips for 15 hours. To identify hybrid cells, co-cultures were trypsinized, replated and checked for double-labeling 12 and 48 hours later.

### Inhibition of Cdc42 and CD44 function

For Cdc42-inhibition, U373 and U87 cells were transfected using Hyperfect reagent (Qiagen) with a mixture of 3 siRNA (small interfering RNA) oligonucleotides against human-Cdc42 sequences (100 nM/each) (these were from 5′-3′: Cdc42-10505, GGCUGUCAAGUAUGUGGAGtt; Cdc42-10428, GGAUUAUGACAGAUUACGAtt; Cdc42-10324,GGGCAAGAGGAUUAUGACAtt, Ambion) or with an Alexa Fluor red-conjugated siRNA, as a transfection control. Inhibited cells were used after 72 hours for seeding on brain slices or after 48 hours for making hanging drops. Secramine-A (Cdc42-inhibitor; kind gift of Dr. T. Kirchhausen, Harvard Medical School, Boston [60]) was added in serum-free medium, at 25 nM, for *in vitro* experiments, and at 15–50 nM in 2% FBS-containing medium for brain slices. To inhibit CD44, we used 1.5 µg/3.5 cm dish of a mixture containing equal amounts of 5 different shRNA lentiviral-plasmids (cDNA-inserts were: CD44-57563, CCGGGCCCTATTAGTGATTTCCAAACTCGAGTTTGGAAATCACTAATAGGGCTTTTTG; CD44-57564, CCGGCCTCCCAGTATGACACATATTCTCGAGAATATGTGTCATACTGGGAGGTTTTTG; CD44-57565, CCGGCGGAAGTGCTACTTCAGACAACTCGAGTTGTCTGAAGTAGCACTTCCGTTTTTG; CD44-57566, CCGGCCAACTCTAATGTCAATCGTTCTCGAGAACGATTGACATTAGAGTTGGTTTTTG; CD44-57567, CCGGCGCTATGTCCAGAAAGGAGAACTCGAGTTCTCCTTTCTGGACATAGCGTTTTTG; all purchased from Sigma), which were co-transfected with GFP-actin. CD44-inhibited cells (with a reduction of CD44 protein level by 27%, which reflects the transfection efficiency of GFP-actin of approximately 30%) were used after 5 days (for slices) and 60 hours (for hanging drops).

### *In vivo* vessel visualization

To label cerebral blood vessels with DiI or DiD (Invitrogen/Molecular Probes), mice (Harlan Laboratories) were anaesthetized with Ketamine (100 mg/kg) and Xylacine (10 mg/kg) and 0.8 ml of a 0.05 mg/ml solution of DiI or DiD (1∶10 dilution of 0.5 mg/ml-DiI/ethanol in 30% sucrose) was injected into the left atrium of the heart, followed 2 minutes later by transcardial perfusion with PBS. To label with ink, anaesthetized mice were transcardially perfused with 15 ml of a 20% solution of black drawing Ink (Pelikan), in Krebs buffer. For intravital experiments, mice were anesthetized with liquid isoflurane (Esteve Veterinaria) and DiI was injected in the retro-ocular venous plexus.

### *In vivo* pericyte labeling

Mouse brain pericytes were labeled *in vivo* using a modified version of a method described by Hirase et al. [61]. Briefly, 0.5 µl of a fluorescent Dextran (Fluoro-Emerald, FlEm, D-1820; Cascade Blue, D-1976; or Alexa-Fluor 647-conjugated [Far Red, FR], D22914; 10 KD, Molecular Probes) (250 mg/ml in 1.5% BSA) was applied to the tip of a hand-pulled heat-sealed glass Pasteur pipette stub (external diameter of approximately 0.5 mm, length of 1.5 cm) and allowed to dry for 20 min at 37°C. For each mouse, two holes were drilled in the skull (1.3 mm in front of the bregma and 2 mm from the midline; 3 mm behind the bregma and 3.5 mm from the midline) and Dextran-labeled glass stubs were inserted into the brain to a depth of 2.5 mm, via pilot holes made using a 28-gauge needle, and left in place for 10 min. This protocol resulted in the strong labeling of the meninges and of a subset of brain pericytes. Mouse brains were implanted with tumor cell aggregates or used for brain slice cultures 5 days after the labeling.

### Preparation of live brain slices and explants

Following *in vivo* labeling of vessels with Ink, DiI or DiD, brains of ICR or NG2DsRedBAC transgenic mice [62], designated as Tg (Cspg4-DsRed.T1)1Akik/J by the Jackson Lab (kind gift from Dr. Dirk Dietrich, Clinic for Neurosurgery, University Clinic, Bonn, Germany), were embedded in 4% low-gelling temperature (LGT)-agarose and cut into 250 µm-vibratome sections or into approximately 1 mm-thick slices, using a razor blade, and collected into ice-cold Krebs solution. Brain sections were incubated with U87 or U373 cell suspensions in a rotating tube for 12 hours, prior to imaging or fixation. Thick slices for grafting were transferred to 3.5 cm petri dishes, held in place with 4% LGT-agarose and equilibrated in culture medium at 37°C for 30 min before cell grafting. Explants for imaging were placed in glass bottom dishes (MatTek Corporation) and secured using 4% LGT-agarose and a glass coverslip. All explants were maintained in α-MEM medium containing 10% FBS.

### Preparation of *in vitro* silicone substrates and co-cultures

Flexible silicone substrates were prepared as previously described [29], [63]. Briefly, 50 µl of silicone oil (viscosity 60,000 cSt, Code 1838, Sigma) was applied to a 12 mm-glass coverslip using a 1 ml-syringe barrel. The silicone-coated coverslip was then centrifuged at 1,000 rpm for 2 minutes in a 12 well microplate (using absorbent paper discs as cushions and to soak up the excess liquid) and then polymerized by heating for 2 seconds over a small yellow Bunsen flame. After 2 hours sterilization with Ultraviolet light (UV), they were finally coated for 1 hour, at room temperature, with 200 µl of human-laminin protein (0.08 µg/ml, Millipore; also used for coating glass coverslips at 10 µg/ml), and finally rinsed with PBS. For co-cultures with GBM cells, pericytes alone were plated on silicone substrates for about 48 hours and imaged by confocal microscopy for approximately 6 hours, to determine the fields for the addition of GBM cells. The same selected fields were imaged for 8 hours after starting the co-cultures.

### Xenografts and thick slice implants

Cell pellets, prepared as hanging drops, were grafted into Athymic Nude mice (Foxn1^nu^, Harlan Laboratories) or into brain slices. Xenografts (1 pellet/mouse) were introduced into the right hemisphere through a small craniotomy (2 to 3 mm from the midline, approximately 1 mm behind the bregma) at 2.5 mm depth, using a stereotactic apparatus and a Pasteur pipette hand-pulled to an internal diameter of 0.38 mm. This produced grafts that integrated into the striatum, the cortex or the hippocampus. Mice, 2-days to 3-months post grafting, were perfused using a mixture of 20% Ink and 4% PFA. Brains were either embedded in agarose and cut at 120 µm using a vibratome, or embedded in paraffin-wax (standard procedures) and cut at 7 µm. For thick slice-grafts, cell pellets were manipulated using tungsten needles and pushed inside the region of the striatum. For grafting into NG2DsRedBAC brain slices, hanging drop cell pellets were injected through the edge of a 1 mm-coronal slice using a hand pulled Pasteur pipette to leave a tumor cell implant extending through the striatum. Slices were fixed between 12 and 72 hours after grafting and cut at 30 µm in wax.

### Western blot, immunocytochemistry, immunohistochemistry and *in-situ* hybridization

For western blots, U373 cell lysates were separated on 10% SDS-polyacrylamide gels, and probed for: human Cdc42 (using mouse monoclonal antibody, BD Bioscience, 1∶350) plus anti-mouse horseradish peroxidase-labeled horse secondary; human CD44 (polyclonal rabbit antibody, Abcam, 1∶4,000) plus anti-rabbit horseradish peroxidase-labeled goat secondary. Rabbit polyclonal (GeneTex) and mouse monoclonal (Sigma) antibodies were used to detect human β-actin (1∶6,000), for loading control. Secondary antibodies were visualized by chemiluminescence (ECL, Amersham) and quantified using Quantity One software (BioRad).

For immunocytochemistry and immunohistochemistry, we used mouse monoclonal antibodies against human-CD44 (1∶100, BD Pharmingen), mouse/human-Cdc42 (1∶50, BD-Transduction Lab), human-Nestin (1∶200, Chemicon) and human-alpha3beta1 Integrin (1∶50, Chemicon); chicken polyclonal against mouse/human-Cdc42 (1∶50, GeneTex); mouse monoclonal against mouse/human-Vimentin (1∶60; clone V-9, Sigma) and alpha Smooth Muscle Actin (αSMA) (1∶70, in vitro, and 1∶50 in thick vibratome slices, Abcam); rabbit polyclonal against Laminin (1∶100; Chemicon), NG2 chondroitin sulphate proteoglycan (1∶80, Chemicon), Nitrotyrosine (1∶50, Millipore) and RFP (1∶400 and 1∶250, in vitro and thick vibratome slices, respectively; MBL); rat anti mouse-CD44 (1∶80, BD Pharmingen), mouse-CD68 (1∶60, Abd-Serotec) and clone rat-401 against mouse-Nestin (1∶150, Millipore). Actin cytoskeleton was visualized by Phalloidin, conjugated with Alexa-488 or 594 (Molecular Probes). Single and double labeling patterns were revealed by fluorescence microscopy, using goat anti-mouse secondary antibodies coupled to Alexa 488 and 594 or to Cy5 (Invitrogen/Molecular Probes, 1∶500 and 1∶250, respectively), goat anti-rat coupled to Alexa 594 or Cy5 and goat anti-chicken coupled to Cy5 (Invitrogen/Molecular Probes, 1∶350). In some cases, fluorescence samples were counterstained with Hoechst (0.001%, Sigma) to label the nuclei and reveal the cytoarchitecture, prior to mounting in Moviol. Alternatively, labeling patterns were revealed by bright field microscopy, using HRP-conjugated anti-mouse secondary antibodies followed by ABC reagents (VectorLabs), plus either Diaminobenzidine (DAB, brown) or 3-amino-9-ethylcarbazole (AEC, red; Sigma). ABC reagents were also used to directly reveal biotin in MiRu-labeled cells, after boiling in citrate-buffer (pH 6). Cytoarchitecture in bright field sections was visualized using Cresyl Violet staining (0.25%). Finally, human nuclei were detected by hybridization with a FITC-labeled human specific pan centromeric probe (1695-F-01 Star*FISH, Cambio, Cambridge UK) and visualized using an alkaline phosphatase coupled-anti fluorescein antibody and BM-purple substrate (Boehringer). *In situ* hybridization for Rgs5 was performed according to standard methods using a full-length mouse cDNA (purchased from imaGenes, Berlin) as a template for the probe.

#### Gene expression analysis on Human Glioblastoma Tumors

Resected Human GBM tumors were analyzed for tumor associated gene expression markers, by mining the Allen Brain Atlas database of *in situ* hybridization data on Glioblastoma tumor sections. An additional Human GBM biopsy was used for immunohistochemistry (provided by Dr. J. Sola, Hospital ‘Virgen de la Arrixaca’ Murcia, University of Murcia, Spain).

#### Ethics Statements

All experiments with mice were in accordance with the Spanish law 32/2007 of November 7^th^, for the care, use, transport, experimentation and sacrifice of animals. The use of human GBM biopsies was approved by the Ethical Committee of Clinical Investigation.

#### Hypoxia detection

To detect hypoxia in mouse brains, mice were injected intra-peritoneally with Pimonidazole-HCL (Hypoxyprobe, Inc) (60 mg/kg body weight) and fixed 30 and 80 minutes later by transcardial perfusion with PFA/Ink. Bound-Pimo was detected in 7 µm-paraffin sections, using a rabbit anti-Pimonidazole antisera (PAb2627) (1∶50), followed by an anti-rabbit secondary antibody conjugated with HRP (1∶150) (both from Hypoxyprobe, Inc), which was then developed with DAB.

### Imaging fixed and live specimens

A TCS-SP2-AOBS laser scanning spectral inverted Confocal Microscope (fitted with temperature and CO_2_ control; Leica Microsystems, Barcelona, Spain) was used for analysis of fixed and live brain explants and for live imaging of cells cultured on glass. For live imaging of silicone substrates, transmission Differential Interference Contrast (DIC) images were collected, simultaneously with confocal fluorescence images. A fluorescence automated DM6000B microscope and a MZ16FA Fluorescence Stereomicroscope (for wide-field microscopy), running Leica Application Suite (LAS) AF6000 Software (version 2.0.2), equipped with a DFC350-FX (monochrome) or DC500 (color) digital cameras (all purchased from Leica Microsystems, Barcelona, Spain) were used to analyze fixed cells, brain explants and histological sections from xenografts. For live imaging of brain explants, we used a TCS SP2 Acousto-Optical Beam Splitter (AOBS) scanning multiphoton system, with an inverted electronically controlled DM-IRE2 microscope (Leica), equipped with temperature and CO_2_ control. For intravital imaging, we used a TCS SP2 RS-scanning multiphoton system with an upright DM LFSA-microscope (Leica), equipped with a special bridge (Bridge 500) housing a mouse head-holder (Luigs & Neumann Feinmechanik und Elektrotechnik GmbH, Ratingen, Germany). For this purpose, a small window was made in the skull over the neocortex of an anesthetized mouse. GBM cells were injected onto the brain surface and the window was then closed with a glass coverslip and held in place with silicone grease. Inverted and upright multiphoton microscopes were connected to either a Millenia-Tsumani or a Mai Tai HP i:Sapphire picosecond laser (SpectraPhysics, Mountain View, CA, USA). All images were collected using internal spectral detectors and LCS Lite Software (Leica). The objective lenses we used were: 20x/0.50 and 63x/0.9 N.A. (both in water) HCX Apo U-V-I; 63x/1.20 N.A. (water) HCX Plan Apo CS; 63x/.30 N.A. (glycerol) HCX Plan Apo CS. For 2-photon and confocal microscopy we used glass bottom-dishes (MatTek Coorporation) and imaged between 2 and 14 hours. Image and video processing (all acquired at 1024×1024 pixel resolution) were performed using LCS-Lite (Leica), Image J-1.41 (NIH Image package) and Imaris Software (x64 7.5.2, Bitplane AG, Zurich, Switzerland). 3D video deconvolution was performed using Blind Deconvolution (algorithm developed by Autoquant, Media Cybernetics, and implemented by Leica). For multiphoton imaging, GFP and DiI fluorophores were excited at 900 nm laser wavelength and the emission filters used were 500/524 and 549/582 nm, respectively.

### Statistical analyses

For statistical analysis, we used Microsoft Excel (2003) and MATLAB Software. Tests were aimed to compare either features and behavior between control and iCdc42 and/or shCD44-treated GBM cells in brain slices or pericyte wrinkling behavior, with and without GBM cells, on deformable substrates. For CD44-interference, we co-transfected shCD44-plasmid with GFP-actin and included only green cells in the analysis. Normal or non-normal distributions were evaluated using Kolmogorov-Smirnov (K-S) test for non-parametric data (one-sample). K–S test for non normal data sets was used for comparing wrinkling behavior of the same region, before and after adding GBM cells, and length of protrusions of GBM cells interacting with blood vessels in brain slices. Length of protrusions (µm) has been grouped as follows: 0(0), 1(0–7), 2(7–14), 3(14–21), 4(21–28), 5(28–35), 6(35–42), 7(42–49), 8(49–56), 9(56–63), 10(63–70). For statistical analysis on protrusion length with siRNA-Cdc42, Secramine-A and shRNA-CD44: controls: n = 15, median = 10, range: 1–50 µm, fraction (0 µm) = 0%; iCdc42: n = 15, median = 0, range: 0–65 µm, fraction (0 µm) = 73%; Secramine: n = 22, median: 0, range: 0–21 µm, fraction (0 µm)54%; controls for Secramine: n = 22, median: 8, range: 2–33 µm, fraction (0 µm) = 0%; iCdc42 shCD44: n = 15, median = 0, range: 0–43 µm, fraction (0 µm) = 87%. K–S was also used for the estimation of the angle of vessel bending in brain slices (angle of bending, in degrees, has been grouped as follows: 0(0), 1(0–20), 2(20–40), 3(40–60), 4(60–80), 5(80–100), 6(100–120), 7(120–140), 8(140–160). For statistical analysis on the angle of vessel bending: controls: n = 13, median = 106, range = 45–125°, fraction bending-angle (0°) = 0%; iCdc42: n = 13, median = 0, range: 0–160°, fraction (0°) = 54%; iCdc42 shCD44: n = 13, median = 0, range: 0–108°; fraction bending-angle (0°) = 62%. For evaluation of bending events: controls: n = 29, total fraction of cell-associated bent vessels = 83%; Secramine A-treated: n = 29, total fraction = 36%. Wilcoxon Mann Whitney test for non-parametric data was used for: vessel co-option in thick slices (n = number of grafts in a total of 14 thick slices). Chi Square (χ2) test for descriptive statistic was used to compare differences in the frequency of vessel bending (bending events) and differences in glomeruloid-like structure formation in brain slices (controls: n = 36, total fraction of cell-associated glomeruloids = 36%; treated: n = 31, total fraction = 6%). Student's t-test for parametric data was used for the number of infiltrating cells on vessels in thick slices (n = number of grafts in a total of 14 thick slices). K–S and t-tests were two-tailed. Significance value (P<0.05) was adjusted to avoid inflated type-1 error: α = 0.05/4 (for brain slices); α = 0.05/2 (for thick slices). Protrusion length was counted only for GFP-actin labeled cells contacting vessels, either directly or through extensions, using Leica Application Suite (LAS) AF6000 software. For statistical analyses on protrusion length, a length = 0 µm was assigned to each cell lacking protrusions. To measure the angle of vessel bending, each bent segment was manually traced from the microscope screen onto an acetate sheet and the angle of deviation, from vessel axis, was measured using a protractor. A bending angle degree = 0 was assigned to straight vessels. For tracking selected wrinkles on silicone substrates, xyzt-data from confocal videos were imported into Imaris Software (5.7 version, Bitplane) and used to generate 4D movies. Identified wrinkles were tracked using the Filament Tracking package on the z-series frame offering the sharpest focus. Specifically, we tracked the displacement of the ends of each wrinkle (designated as e1 and e2), for 5 wrinkles across each area of 0.03 millimeters squared. Among the various statistical parameters offered by Imaris Software for the analysis of each track (e1 and e2), we selected the value defined as ‘track straightness’ (E1 and E2, indicating track displacement/track length), which represents a numerical measurement of how the ends of the wrinkle can move in space and time. With pericytes alone, the track straightness is generally higher than in co-culture with tumor cells. This reflects the tendency for wrinkles to either: 1) drift laterally or drift laterally and pivot, which represents the majority of the pericytes located in the regions surrounding the nodes and in the regions connecting them (internodes); 2) increased local movement and/or less lateral displacement, shown by a relatively small percentage of pericytes located in the anti-node regions. In the presence of tumor cells, the reduction in the wrinkle track straightness indicates the second behavior as the main modality of pericyte contraction. Imaris and Leica Application Suite programs were used for cell measurements and quantification in brain explants and on silicone substrates. The 3D-scatter plot used to illustrate wrinkle data distribution was obtained using MATLAB Software.

## Supporting Information

Figure S1 **Vessel co-option and remodeling by GBM cells in brain slices.** GFP-actin transfection of GBM cells allowed to investigate the actin cytoskeleton dynamics during tumor cell/vessel interaction. **A**, *In vitro,* phalloidin staining (red) of glioblastoma cells transfected with GFP-actin construct (below) shows complete overlapping (yellow) with the actin cytoskeleton (green), at intracellular level (stress fibers, white arrowhead) and in cellular protrusions, both in ruffles (white arrows) and in filopodia (arrow in inset), while GFP transfection alone shows very little co-localization in ruffles (white arrow and yellow color) and no co-localization either in stress fibers (arrowhead) or filopodia protrusions (arrow in inset). **B**, 2-photon video frames showing a co-opting glioblastoma cell making initial contact with a vessel (DiI-red) through cell polarization and emission of actin-enriched extensions (arrowheads); the white dotted line indicates the absence of protrusions at t0. Longer extensions with discontinuous actin (arrows) are polarized towards another vessel (red dotted line). **C**, Cell co-option of mouse brain meningeal vessels, following intracranial injection of GFP-actin labeled-GBM cell suspensions. Intravital imaging of the superficial neocortex confirms that injected U373 tumor cells (also labeled with CMTMR, red), after initial polarization towards blood vessels (v, DiI, red, dashed lines), emit actin-enriched thin cellular extensions (white arrow in i), which contact the vessel abluminal surface (inset: beaded organization of actin in the protrusion, arrows). Although thicker protrusions are detectable (yellow arrows in ii and iii), they always bear thinner terminal elongations that contact the vessel (dotted lines in magnification, iii). **D**–**E**, frames from two 4D rendered-confocal videos (in **E** only the vessel is rendered), showing U373 cells modifying blood vessels (Ink-filled, grey) in brain slices. **D**, an additional example of a flectopodia-linked vessel modification (yellow arrows and lines); white arrows point to moniliform actin-distribution in flectopodia. Another, less elaborate, type of local vessel modification is also observed (**E**, live, and **F**, fixed; yellow arrowheads in **E**–**F** and yellow lines in magnified insets in **E**), in which a cell envelops and kinks a narrow vessel, as indicated in the scheme (**G**). This type of local vessel alteration is coupled to the retraction of a long GBM cellular extension (**E**, white arrows) and formation of subcortical actin fibers (yellow arrow). **D** and **E** are taken from sequential videos of the same cell, with an interval of 1 hour (red arrows: vessel previously bent in **D**). Time in minutes. Scale bars: 6 and 1.5 µm (**A** and insets), 10 µm (**B**, **D**), 20 and 11 µm (**C**-i and **C-**ii), 9 µm (**F**).
(TIF)

Figure S2 **GBM cells specifically target brain pericytes** ***in vivo*** **and alter their contractility** ***in vitro*** **. A**, Experimental scheme showing insertion of GBM cell-pellets into the striatum of NG2DsRedBAC-brain slices. 3D reconstructions from both a confocal video (**B**) and fixed samples (**C–D**), illustrating U373 cells (labeled either with FR-dextran/GFP-actin or FlEm Dextran) during (**B**) and after (**C–D**), respectively, the co-option of vessels (arrowheads, DiD-blue; already highly modified in **C** and **D**) at the graft/host border (dashed lines). White arrows point to GFP-actin-labeled flectopodia. Asterisk in **C** indicates red NG2^+^-cells recruited into the graft. Boxed area in **B** is magnified in Fig. 2A. **E**, 3D rendering of the boxed region in **D**, showing an incipient glomeruloid blood vessel (arrowhead), resulting from the interaction of GBM cells (green, white arrows) with NG2^+^-perivascular cells (red, bounded by dashed lines). Cell nuclei in blue (Hoechst, **C–F**). **F**, 3D rendering showing another example of a GFP-labeled GBM cell (green, white arrow) in contact with a NG2-labelled pericyte (black arrowhead) on a bent vessel segment (outlined by dashed lines), in brain slices. Yellow asterisk illustrates partial sharing of cytoplasm. **G**, Confocal section-video frames of a MiRu^+^, GFP-actin^+^-U373 cell seeded 8 hour earlier, showing the co-transfer (white arrowheads in magnifications of boxed area) of GFP-actin (green) and MiRu-Dextran (red) into a DLP (Cascade Blue, CB, blue). Note the similarity with the double labeled cytoplasm in the dashed areas in **E**. **H**, Purified brain pericytes grown *in vitro* were analyzed by immunocytochemistry for the markers indicated (in some cases were pre-labeled with FlEm-Dextran, green, or after challenge with 1 µm-fluorescent latex beads (FLB) to test for phagocytic uptake). **I**–**K’**, Heterogeneous distribution of actin proteins (phalloidin, green, in **i** and αSMA, red, in **I**–**J**) in pericytes plated on silicone plus human laminin. Wrinkles in magnified box (arrowheads, **K’**) are strongly correlated to αSMA expression (Ref [64] in Methods), as indicated in **K**. (**L**) Coronal section through the striatum (Str) of a brain pre-labeled for DLPs and perfused with black-Ink shows that DLPs (**M**, green, asterisks) express αSMA (magenta, arrowhead in magnification in **N**), which correlates with constricted segments (**N’**, yellow arrowhead) of a Ink-filled vessel (**N’**, white arrowhead). Nuclei (Hoechst) are in blue. **O**–**P”**, The dramatic effect of GBM cells (FR dextran, magenta) on pericyte contraction is illustrated by comparing wrinkling patterns from confocal videos of the same field, recorded before (**O**) or after (**P**) GBM cell addition (host/tumor border indicated by dashed line in **P**). Asterisks (red in **O**) indicate the positions of 3 nodes, two of which (yellow in **P**) are destroyed. In the presence of GBM cells, destabilization of the wrinkles along the margin (replacement of stable pre-existing wrinkles, red arrows in **O’**, by unstable wrinkles that come, white arrowheads, and go, yellow arrowheads, in **P’**) correlates with dynamic protrusion and retraction of GBM cell flectopodia-like extensions (indicated by dashed arrows in **P’**). **O”** and **P:** show the tracking data illustrated in Figure 2H projected onto the original, initial time point for each trace (t2 and t14, respectively). Time in minutes. Scale bars: 30 µm (**B**–**D**, **M**, **O’**, **P’**), 10 µm (**F**–**G**, **N**), 25 µm (**H**), 30 µm (**I**), 100 µm (**O**–**P**), 25 µm (**O”**–**P”**).
(TIF)

Figure S3 **Cdc42 protein localizes in flectopodia varicosities and is transferred into pericytes in xenografts. A**, Confocal video-frames of a U87 GBM cell *in vitro*. Co-transfection with GFPactin (green) and CherryCdc42 (Ch-Cdc42, red) reveals the striking dynamic association between actin beads and local accumulations of Cdc42 (white arrowheads in magnifications) in the cytoplasmic varicosities within the long cellular extensions (white arrows in **A**). Arrowheads (**B**) and dashed-arrows (**C**) indicate varicosities positive for GFP-tagged outer mitochondrial membrane protein (GFP-RR) and β3-integrin (arrowheads in insets), respectively, in the extensions of GBM cells cultured as 3D aggregates on laminin-coated glass (confocal video frames). Asterisk points to a labeled fragment released from the tip of the extension. Time in minutes. **D**, 3D confocal reconstruction (100 µm section) of a 2-day Ch-Cdc42-U373 cell xenograft, immunostained for the Cherry-tag (red); dextran-labeled-host cells, cyan. th, thalamus; hip, hippocampus; men, meninges. **E**, Graft/host interaction-zone from an adjacent section to the boxed-area in **D**, magnified in **F**; white arrows point to a cell double-labeled for Ch-Cdc42 and host-dextran. **G** shows localized tumour-Cdc42 transfer into a dextran-labeled, CD68^–^host cell (white arrows), indicating that cell/cell transfer can occur by a mechanism other than phagocytosis. White arrowheads point to a CD68^+^-dextran labeled-phagocyte. **G** is an adjacent section in the region boxed in **D**. Co-localizations (in **F** and **G**) are confirmed in confocal xyz-sections (white asterisks). Scale bars: 8 and 2 µm (**A** and magnification), 25 µm (**B**), 15 µm (**C**), 550 µm (**D**), 50 µm (**E**), 20 µm (**F**), 10 µm (**G**).
(TIF)

Figure S4 **GBM cell-flectopodia modify local wrinkling patterns of pericytes plated on flexible substrates.** Videos frames (combined DIC and fluorescence confocal microscopy) of GBM cell/pericyte co-cultures on laminin-coated silicone substrates. **A**, Flectopodia extending from a FarRed-labeled U87 cell (arrows) coincide with the presence of new wrinkles (arrowheads). **B**, Wrinkles, centered around terminal varicosities (white arrowheads) of the flectopodium (white arrow) extending from a MiRu-labeled-GBM cell (red color shown in first and final frames, only), are induced with a period of approximately 40–50 min (red arrowheads: presence, yellow arrowheads: absence). **C**, Flectopodia of two MiRu^+^-GBM cells (arrowheads in **C”** [lower magnification of **C–C’**]), one of which transfected with GFP-actin (green, white arrowheads in **C** and **C’**; light blue arrowheads in **C**”), show local accumulations of GFP-actin and dextran. Note that the waves of altered contraction of the substrate (red/yellow arrowheads in **C**) are in phase with periodic enrichment of actin at the tip of the flectopodium (white arrowheads in **C**–**C’**; blue arrowheads in **C”**). **D**, Cytoplasmic particles (1 and 2, indicated by green dots), either pre-existing (magenta arrowheads) or released as varicosities (dark blue arrowheads) from the tips of flectopodia (arrow) translocate and divide within the target pericyte. Time in minutes. Scale bars: 40 µm (**A**, **D**), 20 µm (**B**), 25 µm (**C**).
(TIF)

Figure S5 **Tumor cell/host pericyte interaction generates fusion-like hybrids both in xenografts and brain slices. A**, Co-opted blood vessels (white and black-filled white arrows), in the graft 7-days post-implantation. **B**, MiRu^+^-perivascular cells (arrowheads) inside the graft (i) are negative for human-centromeric DNA (h-cen DNA) (i’) and positive for mouse Rgs5 (I”), suggesting potential fusogenic zones in which human tumor cytoplasmic determinants are mixed with mouse nuclei. **C**, Scheme showing two FlEm (green)-injection sites, used to pre-label brain pericytes (DLPs) and the position of the GBM graft (MiRu-labeled, red). Dashed line indicates the plane of section in **D**, **E**, **F** and **J**. **D** and **E**, Combined bright field and fluorescent images indicating a 3D confocal reconstruction of 120 µm-vibratome sections through a U373 (**D**) and a U87 (**E**)-graft, respectively, showing co-opted blood vessels in the tumor core (Ink-filled; white arrowheads). cp, choroid plexus. (**D**, **E**, **I**) FlEm^+^ and MiRu^+^-GDH cells (arrows in **D**, **E**; arrowheads in **I**, magnification of an adjacent section, at the position indicated in **D** and **E**) on a constricted (**I**, arrow) and dilated (**I**, thicker arrow) vessel segment (grey color). Cell nuclei in blue (Hoechst). **F**–**G**, A combined bright field and fluorescent image showing DLPs from a control brain (arrows in **F**; arrowheads in **G**), located on straight vessels (Ink-filled, black in **F**; grey in **G**). Central panel in **G** shows lack of MiRu (red) signal. **H**, 3D rendering confirms that DLPs are pericytes, based on their perivascular location and large nucleus (arrow), compared with the smaller size of an endothelial cell nucleus (arrowhead). **J**, Staining for h-centromeric DNA of a 7-day MiRu^+^-U373-xenograft, grafted into an unlabeled host. Black arrows in low magnification point to cells magnified in **K** and **L**, whose nuclei are either strongly positive (**L**, white arrows, type-1 cells) or negative (**K**, black arrows, presumptive GDH cell) for h-cen DNA. Another putative GDH cell is coupled to infiltrating type-1 cells (black arrows in **L**). Equivalent fields are shown as fluorescence (left hand side; MiRu, red, and Hoechst, pale blue) and bright field micrographs (right hand side); vessels: v, ink-filled, black. Type-1 cells (**N**, arrowheads) express h-CD44 (green), which is absent in the presumptive GDH cell (**M**, white arrowheads); ve: ventricle; v: vessel; yellow arrowheads: vessel dilations; white arrow: vessel constriction. **O**, Table showing the labeling characteristics and phenotypes for cells located in the tumor core (Type-1fusion-like, Type-1f) and at the infiltrating margin (Type-1, Type-2 and GDH), for both U373 and U87 GBM xenografts. **P–R**, Confocal pictures of brain slices, pre-labeled with FlEm-Dextran for pericytes (green) and seeded with MiRu^+^-U373 cells (red). Examples of novel recombinant cell types, that resemble those found in mouse xenografts: **P** shows a type-1 like-cell located on a blood vessel (yellow arrow) and associated with a FlEm^+^-particle (white arrowhead in magnification); type-1f-like cells are also present, either closely apposed (arrowheads in **Q**) or partially co-labeled (white arrow in the confocal section, and asterisk in inset). **R**, Double labeled cells (FlEm^+^ and MiRu^+^), similar to GDH cells, are also visible. Arrows point to the almost complete co-localization of the two Dextrans. Vessels: Ink-filled (white) or outlined by dotted lines; nuclei, Hoechst, blue. Scale bars: 100 µm (**A, D**, **J**), 30 µm (**F**), 20 µm (**G**), 8 µm (**H**), 10 µm (**B**, **K–N**), 7 µm (**P**–**R**).
(EPS)

Figure S6 **Markers of altered pericyte contractility in short and long-term GBM mouse xenografts and induced hypoxia. A** and **C** Show triple-labeled confocal reconstructions of 120 µm-coronal sections from 7-day MiRu-labeled (red) U373 (**A**) and U87 (**C**) tumors, grafted into mice with pre-labeled DLPs (FlEm, green) and stained for αSMA (cyan). Although αSMA, a marker of contractile pericytes, is generally more prevalent in perivascular cells in U373 than U87 grafts (white arrowheads, **A**–**D**), both cell lines (**E** and **F**, boxed areas in **A** and **C**) express very high levels of αSMA (arrowheads in **E**–**F**) in GDH fusion-like hybrids (arrows), located outside the graft on highly modified vessels (Ink filled, black). Similarly, Nitrotyrosine, a marker of nitrative stress, is also present in the grafts (arrowheads in **H** and **J**), but shows its highest level (arrowheads in 1–1′) in association with αSMA^+^-GDH cells (arrows), accumulating either within the GDH cell itself (**G**1, 3) or in immediately adjacent pericytes (**I**1’, 3′). This differential localization of Nitrotyrosine between U373 and U87 is also evident in the tumor core, where it is found either in putative fusogenic zones at the tumor/host interface (**G**2) or in contact with MiRu^+^-cells in perivascular locations in the graft core (**I**2’), respectively. Infiltrating tumor cell-tongues (**K**, MiRu, red, white arrows) and Pimo-stained hypoxic regions (**L**, brown, Pimonidazole) in a 7-day U373-xenograft. White and black dotted lines: core of the implant; red dotted line: hypoxic penumbra in **L**. GDH cells are indicated by light blue arrowheads in **K** and by white arrowheads in magnifications of the inset (**M**). Modified vessels in **M** (magenta arrowheads), associated to GDH cells, connect directly to the most intense hypoxic zone (red arrows in **K** and **L**). White and black arrowheads (**K** and **L**) show a ring of Pimo staining, surrounding degenerating material in the tumor core. Yellow arrowheads in **K** point to type-2 cells on meninges. A continuing role for GDH cells in regulating vascular tumor physiology and in creating a hypoxic environment at the expanding tumor co-opting front is supported by their presence in long term U87 and U373 xenografts (arrows in **P** and **T**, which are magnifications from sections adjacent to those stained with Nissl, **N**–**O**, and **R**, respectively) and their expression of αSMA (arrowheads). Note that GDH cells (**P**, **Q** and **T**, **U**) are a common feature of the GBM graft margin, despite the apparent different mode of expansion of U87 and U373 tumors: a smooth defined boundary inside the sub-ventricular zone (**N**–**O**) versus an interdigitating boundary within the parenchyma, respectively (arrows in **S**). Arrow in **O** points to a disrupted boundary zone in U87, in close proximity to a GDH cell (**P**). Furthermore, these fusion-like hybrids (white arrows) show accumulation of Cdc42 protein (white arrowheads) at vessel modification sites (modified vessels are outlined by dashed lines) in both 7-day (**V**–**W’**) and long term (**Q** and **U**) U373 and U87 cell xenografts, respectively. Interestingly, Cdc42 is also detectable in subcellular fragments located both within abnormal dilated vessel segments in 7-day grafts (yellow arrowhead in **X**) and in large sinusoids (white arrowheads in **Y**) in the core of 1-month tumors, in close proximity to either GDH cells (yellow arrows in **X**) or strongly positive Cdc42-perivascular cells (white arrow in **Y**), respectively. Scale bars: 160 µm (**A**), 15 µm (**E**, **F**), 150 µm (**G**), 50 µm (**G**-1, **I**-1′, **Q**), 25 µm (**G**-2), 20 µm (**I**-2′), 4 µm (**I**-2′-magnification, **G**-3, **I**-3′), 160 µm (**K**), 10 µm (**M**), 1 mm (**N**), 100 µm (**O**, **S**), 10 µm (**P**, **T**, **U, V**, **W**), 8 µm (**X**), 40 µm (**Y**).
(TIF)

Figure S7 **Generation of pericyte/GBM cell fusion-like hybrids** ***in vitro*** **.** Fluorescence micrographs showing the presence of double labeled-progeny (arrowheads in **A** and **C**), 48 hours after co-culturing FlEm (green)-pericytes with GBM cells, labeled either with MiRu (MR, red, **A**) or Cherry-tagged Cdc42 protein (red, **B**–**C**). Arrows indicate in **A** parental-type single labeled-cells and in **B** plurinucleated (black-filled white arrowheads), putative fusogenic zones. Immunocytochemistry on double-labeled derivatives shows that two human GBM markers associated with stemness (h-Nestin and h-CD44, **D** and **E** respectively) are maintained in early stages (12 h after replating, white arrowheads). Particularly striking is the range of double-labeled cell morphologies seen at 12 h, which includes cells containing both an enormous macronucleus (yellow arrowhead in **D**) and multiple micronuclei (dashed arrow), each surrounded by local accumulations of co-labeled cytoplasm, suggesting both abnormal DNA replication and possible segregation of aneuploidy mini-cells. Furthermore, the differential distribution of human stem markers in the fusion-like derivatives suggests that a highly asymmetric process of cytoplasmic segregation occurs rapidly following mixing (**E**). In addition to complete cytoplasmic co-labeling, we also found cell/cell pairings (arrowheads in **F** point to double-labeled zones) that appear to involve flectopodia-like extensions (arrows). Asterisk: accumulation of α3β1 integrin at the tip of the flectopodium. Nuclei, Hoechst, blue. Scale bars: 30 µm (**A**), 15 µm (**B**, **C, E**), 40 µm (**D**), 20 µm (**F**).
(TIF)

Figure S8 **CD44 synergizes with Cdc42 during GBM cell co-option and modification of blood vessels in brain slices. A**, GFP-actin transfected tumor cells (green) treated with the Cdc42-inhibitor Secramine-A appear morphologically round and not polarized (white arrowhead), on a straight vessel (black-filled white arrowheads). **B**, The graph shows the effect on vessel modification due to the inhibition of Cdc42 activity by Secramine-A. Controls, n = 29; Secramine, n = 42. **C**, Front and side views (**C** and **C’**, respectively) from the 4D rendering of a confocal video, showing a wild type-U373 cell implant (MiRu, red) next to an iCdc42 (FlEm, green)-treated implant. Wild-type cells (arrows) exit the graft on blood vessels (grey), while iCdc42-cells (arrowheads) are found scattered in the parenchyma. **D**, High level of CD44 (blue) at contact sites with bent vessel segments (red arrowheads) in GFP-actin-transfected GBM cells (white arrows). Double inhibition for CD44 and Cdc42 leads to a round cell phenotype (GFP-actin^+^, green in **E**; CD44 in blue). Inset shows lack (red dotted-line) of CD44-labelling (grey color), with no evidence for vessel bending (yellow arrowhead). **F**–**H**, Statistical analysis of the outcome of Cdc42 interference, coupled to down-regulation of CD44, shows stronger inhibitory effect compared to Cdc42 inhibition alone; **F**, controls and iCdc42 shCD44, n = 15; **G**, controls and inhibited, n = 13; **H**, controls, n = 36; treated: n = 31 (asterisks: see Methods for length/angle grouping). **I**–**J**, Quantitative effects of Cdc42 and CD44 interference (graph, left hand side) on the frequency of 5 tumor cell morphologies in brain slices (hypothetical temporal sequence, right hand side). Scale bars: 10 µm (**A**, **C**–**D**).
(TIF)

Figure S9 **Inhibition of Cdc42 in GBM cells converts contractile pericytes into a phagocytic, macrophage-like phenotype on silicone substrates and leads to tumor suppression** ***in vivo*** **. A**–**H”**, To provide a sketch of the global dynamics of GBM cell/pericyte interactions, we created a single 2D image (maximum projection in both z and time axes) for each 4D movie of pericytes alone (**A**, **C**, **E**, and **G**; DIC optics) and co-cultures, using FarRed-labeled (FR)-GBM cells, either U87 (**B**–**B”**), iCdc42-U87 (**D**–**D”**), U373 (**F**–**F”**) and iCdc42-U373 (**H**–**H”**) (**X**, **X**’ and **X**” represent DIC, DIC plus fluorescence and fluorescence alone, respectively). This analysis showed a striking difference between the behavior of the two cell lines. U87 cells occupy a contained region of the field, with a clearly discernable boundary (dotted line in **B”**), reflecting a random cell movement within a circumscribed area. In contrast, U373 cells produce more or less parallel lines distributed throughout the field (arrows in **F”**), which represent the trajectories of directed cell migration. Red arrowheads: node regions present in the field, before and after GBM cell addition. Wild-type GBM cells also cause the activation of flat and contractile pericytes (P^f^) into raised (P^r^) and spheroidal pericytes (P^s^, green filled-black arrowheads in **B**–**B’** and **F**–**F’**), normally characteristic of plastic anti-node regions of pericytes plated alone (tabulated in **M**). P^s^ pericytes also include mitotic and pseudo-mitotic cells (those with a transient cleavage furrow). The analysis of 4D-2D projection patterns for GBM cells inhibited for Cdc42 showed that the overall field features for both U373 and U87 cells were completely transformed. First, the normal organization of U87 as a circumscribed block and of U373 in quasi-parallel lines is replaced, in each case, by discrete compact FR-cell patches (**D”** and **H”**, respectively), reflecting locally confined, rotating trajectories. Second, we found an overall reduction in wrinkling activity, correlated with a further activation of pericytes into a macrophage-like, phagocytic phenotype (P^s^/M, blue-filled black arrowheads point to one example), which come to surround the iCdc42-GBM cells (dotted lines in magnifications **D”’** and **H”’**). **I**–**J**, Two examples of tumor cells (yellow arrows) been killed and engulfed by activated, macrophage-like pericytes (white arrowheads in **I**). Double arrows indicate end-points in the phagocytic process. Note the morphological transformation of an initially flat pericyte (P^f^ in **J**) into a spheroidal, macrophage-like phenotype (P^s^/M), via a transient raised state (P^r^). Time in minutes. **K**–**L**, Single time point video-frames, indicating that the appearing of round, macrophage-like pericytes in the top-half of the field (blue-filled black arrowheads in **L**) is coupled to the disappearance (yellow arrowhead) of a node (red arrowhead in **K**). A second node (red-filled black arrowhead) is established subsequently in the bottom-half of the field, where no GBM cells are visible. **M**, Tabulation of activated pericyte phenotypes (number of pericytes from a total of 600 cells/millimeter squared). **N**-**O**, Quantification of Cdc42 and CD44 knock down efficiency by Western blot and immunohistochemistry. Western blot (**N**) shows Cdc42 and CD44 levels (reduced to 20% and 73%, respectively), normalized for actin across different experimental groups. **O**, Histological and immunohistochemical analysis of control or iCdc42-U373 cell pellets pre-implantation, using the markers indicated. **P**, Cresyl Violet staining of U373 graft/host boundary (7-day xenografts), labeled *in vivo* for blood vessels (black Ink). Red arrow (**P**i) indicates abnormally dilated vessels; 1, boxed area in **P**i, showing the infiltrating margin of a control-graft (red dotted line); red arrows in 2 (boxed area in **P**1) point to dilations and constrictions of a vessel colonized by tumor cells. Boxed areas in **P**i’ show, respectively, the well-defined margin (dotted line in **P**1’) and a morphologically normal vessel (red arrows in 2′), from an iCdc42-graft; c, cortex; cc, corpus callosum. **Q**, Vimentin labeling (green) of the tumor mass in wild type-grafts (arrow in **Q**i and magnified box-1) and of the host microglia (arrowheads in **Q**i and **Q**1). iCdc42-grafts appear almost negative for vimentin (arrow in **Q**i’), while there is an increase in host microglia response (arrowheads in **Q**i’ and in magnification of box-2), which spread through the corpus callosum (small red arrow). Scale bars: 100 µm (**A**–**H”**, **K**, **O**–**Q** and magnification), 20 µm (**I**, **J**).
(TIF)

Figure S10 **A synexpression group of perivascular contractile/hypoxia cell markers in human glioma. A–C**, Three independent glioblastoma tumors, taken from the Allen Brain Atlas database (www.brain-map.org). Note co-expression of the markers indicated (arrows and arrowheads in magnifications) in abnormal tumor vasculature (small arrows in Haematoxylin and Eosin stainings, HE), located at the border between the least (strongly stained) and more (weakly stained) differentiated tumor tissue (shown as dotted lines in **A**). The comparison of a presumptive fusion zone in the core of a 7-day U373-xenograft (MiRu^+^, **D**) with an anomalous vascular structure in a human GBM biopsy (**E**) shows the similarity of marker expression around highly modified vessels, raising the interesting possibility that the hypertrophic Cdc42^+^, laminin-rich-perivascular regions are the sites of ongoing GBM cell/pericyte amalgamation (white arrows in **D**; in **E** white arrows point to endothelium-free, hypertrophic perivascular layer). Dotted lines indicate the lumen of abnormal vessels; endothelial cell nuclei (**E**-iv, small white arrows) are visible only in that part of the vessel-wall lacking Cdc42 (white arrowheads). Nuclei in blue, Hoechst. Scale bars: 17 µm (**D**), 40 µm (**E**).
(TIF)

Figure S11 **Co-option signals 1 and 2 at the tumor/host margin and their hypothetical mediators.** **A**, Interaction at the host/tumor margin. In the presence of functional Cdc42 (**B**), both signal-1 and 2 are active. Signal-1 activates pericytes to trigger the innate immune system and phagocytic response. Signal-2, by co-opting naïve, contractile pericytes, leads to vessel co-option and remodeling, and acts as an immunosuppressor by limiting the number of naïve pericytes available to be converted into macrophage-like cells. Signal-1 plus signal-2 also leads to the production of fusion-like hybrids. In the absence of Cdc42 (**C**), the constraint on pericyte activation is relieved, leading to rapid tumor clearance. **E**, Hypothetical scheme showing putative recombinant cell types generated by flectopodia-mediated transfer into naïve pericytes (TP1, TP2, left hand side), or by resolution of the tumor cell/pericyte-‘fusion hybrid’ (Hy1–Hy4, right hand side). The phenotype of the resulting cells is hypothesized to be specified by the independent segregation of cytoplasmic and nuclear determinants, where the behavioral phenotype depends primarily on the cytoplasmic factor. (**D** and **F**) Key legends. **G**, Signal 1 could involve pro-inflammatory molecules (red) released by GBM cells (green), which activate contractile pericytes to a macrophage-like, oncolytic phenotype (M1-type, white). During this acute inflammatory process, extracellular ATP (e-ATP), for instance, could promote the M1-type polarization and assembly of the P2X7R/NLRP3 inflammasome [64]. Signal 2, which opposes signal 1 and requires GBM cell/pericyte contact through flectopodia, results in the altered contractility of the pericyte and the localized clustering of F-actin in the pericyte cytoplasm (Figure 3**F** and Figure S11 **H**). This clustering could be due to the flectopodia-dependent transfer of F-actin beads into the target pericyte. Alternatively, or in addition, it may involve the production of PPi, from extracellular ATP [64], [65]. ATP hydrolysis involves the activity of the ectonucleotidases CD39 and CD73, present on pericytes [66] and GBM cells [67], respectively, in combination with the E-NPP1, which is enriched in glioma stem cells [68]. F-actin clusters could in turn inactivate the inflammasome and convert macrophage-like pericytes from M1 (inflammasome active) to M2-like type (M2?) (uncoupled P2X7R/NLRP3, inflammasome inactive). ATP hydrolysis also produces Adenosine, which promotes immunosuppression [69] and cell fusion [70]. Signal 2, therefore, may act through F-actin clustering and ATP hydrolysis to prevent the formation of M1-type macrophage-like pericytes and instead maintain a M2-type macrophage-like phenotype, thus supplying a chronic, low level inflammation-state which can never properly resolve.
(EPS)

Movie S1 **Flectopodia mediate large-scale vessel re-arrangement.** 4D-reconstruction of a two-photon video, showing GFP-actin U373 cell-flectopodia (green) with beaded actin (white arrows) inducing a vessel re-arrangement (red, DiI; white arrowheads, bent vascular structures). GFP-actin transfected cells are also labeled with CMTMR (red). Another GFP-actin-cell (yellow arrow) co-operates in translocating modified vessel segments. Yellow arrowheads, actin beads in the initial cytoplasmic bridge (initial blue arrows); red arrows, fusion of the first cell-flectopodia; magenta arrow, coalescence between the pre-existing flectopodia and the leading edge of the second cell; final blue arrows, cytoplasmic bridge with reverse flow of actin beads after merging of the two cell extensions. Δtime: 6 minutes; time, 6 hours and 18 min; rate: 4 fps (frames per second).
(AVI)

Movie S2 **Tumor cytoplasmic transfer into DLPs and vessel modification.** Four-dimensional reconstruction from confocal video, showing the MiRu**^+^** cytoplasmic extension of a U373 cell (red) transferring MiRu**^+^** material (white arrows) into a FlEm**^+^** pericyte (DLP, green), at the same time that adjacent vessels (Ink-filled, grey color) are modified (green arrows). Δtime: 6 minutes; time: 1 hour 30 min; rate: 2 fps.
(AVI)

Movie S3 **Isolated brain pericytes show contractile activity on laminin-coated silicone substrates.** Flat pericytes generate parallel moving-wrinkles (red arrowheads, in the regions delimited by the red dashed lines), organized around highly contractile nodes (red arrows). Yellow dashed lines outline the internode regions where wrinkles move and pivot (yellow arrowheads), while the green arrowhead points to a raised, non-wrinkling, activated pericyte in an anti-node region (approximated by the green dashed boundary). Δtime: 6 min 30 sec; time: 1 hour 30 min; rate: 3 fps.
(AVI)

Movie S4 **GBM cells modify the contractility of pericytes plated on laminin-coated silicone substrates.** The area occupied by Far-Red (magenta color) dextran^+^-U87 cells is delimited by the yellow dashed line (analogous to a graft/host margin). Tumor-flectopodia (white arrows) are associated with modifications of the wrinkling substrate (green arrowheads). Red arrows and red arrowheads indicate flectopodia extensions emanating from groups of GBM-cells, at progressively later times. The altered position of the yellow dashed-line shows the modification of the co-opting-tumor front (first and final positions are compared in the final video-frame). Yellow arrows indicate the difference in the pericyte wrinkling activity at the interacting front. Δtime: 10 minutes; time: 8 hours; rate: 3 fps.
(AVI)

Movie S5 **Actin-enriched flectopodia are associated with modifications in pericyte contractility.** Confocal video showing GFP-actin-U87 cells (green) interacting with pericytes plated a on a laminin-coated silicone substrate. The contact of actin-enriched extensions of a GBM cell with the substrate (white arrows) is linked to new wrinkle-formation (red arrows), indicative of altered pericyte contractility. Yellow arrows point to substrate-relaxation following the retraction of the cell extension. Cyan arrows point to a GFPactin^+^-cytoplasmic fragment (which varies between 3.6 and 4 µm in diameter, lines indicate the trajectory), moving onto the substrate in tight contact with pericytes. Δtime: 7 minutes; time: 2 hour 30 min; rate: 2 fps.
(AVI)

Movie S6 **The contact with pericytes on silicone substrates is mediated by GBM cellular extensions, which can release cytoplasmic fragments.** Magnification of a detail from Video 4. While moving on the top of contractile pericytes spread on laminin-coated silicone substrates, a FR^+^-U87 cell contacts the underlying pericytes through highly polarized protrusions (red arrows), and releases cytoplasmic fragments (asterisks) which are visible as varicosities (white arrowheads) at the tip of the retracting extensions (white arrows). Δtime: 10 minutes; time: 1 hour 10 min; rate: 1 fps.
(AVI)

Movie S7 **GBM cells merge with brain pericytes on laminin-coated silicone substrates.** Magnification of a detail from Video 4, showing the step-by-step sequence of a merging between a tumor cell and a pericyte. The FR dextran^+^-cytoplasm from a U87 cell (white arrowheads) is progressively transferred (yellow arrowheads) into an adjacent unlabeled pericyte (white arrows). Asterisks indicate the merging cell product, which then migrates away. Δtime: 10 minutes; time: 1 hour 40 min; rate: 1 fps.
(AVI)

Movie S8 **GBM cells are destroyed by activated phagocytic pericytes.** An iCdc42-U87 GBM cell (yellow arrows) is pursued and caught by a non-contractile, macrophage-like, activated pericyte (white arrowheads), which appears to contain cytoplasm from a previous engulfed FR^+^-GBM cell (yellow arrowheads). Blue arrowheads indicate the sequence of enveloping and destruction of the tumor cell, with the resulting uptake of the residual GBM cell into the phagocytic cell-cytoplasm (blue arrowheads). A second activated pericyte (white arrows) participates in the immune reaction. Δtime: 10 minutes; time: 8 hours; rate: 3 fps.
(AVI)

Movie S9 **Inhibition of Cdc42 activity (iCdc42) in GBM cells induces pericyte immune response on laminin-coated silicone substrates.** An iCdc42-U373 GBM cell (yellow arrows) is chased by an activated host pericyte (white arrowheads), which finally engulfs it (blue arrowheads). The result of another phagocytic event is shown in the upper part of the video (second blue arrowheads). Note that the entire field is characterized by dendritic-like cells connected in a network by long thin extensions (red arrowheads). Δtime: 10 minutes; time: 8 hour; rate: 4 fps. Time between frames: 10 min; total time: 8 h.
(AVI)

Discussion S1 **Tumor cell/pericyte fusion.**
(DOCX)
