# Supplementary figures and images for: Glioblastoma: A Pathogenic Crosstalk between Tumor Cells and Pericytes
Source: PLoS One. 2014 Jul 17;9(7):e101402. doi: 10.1371/journal.pone.0101402 (PMC4102477; doi:10.1371/journal.pone.0101402)

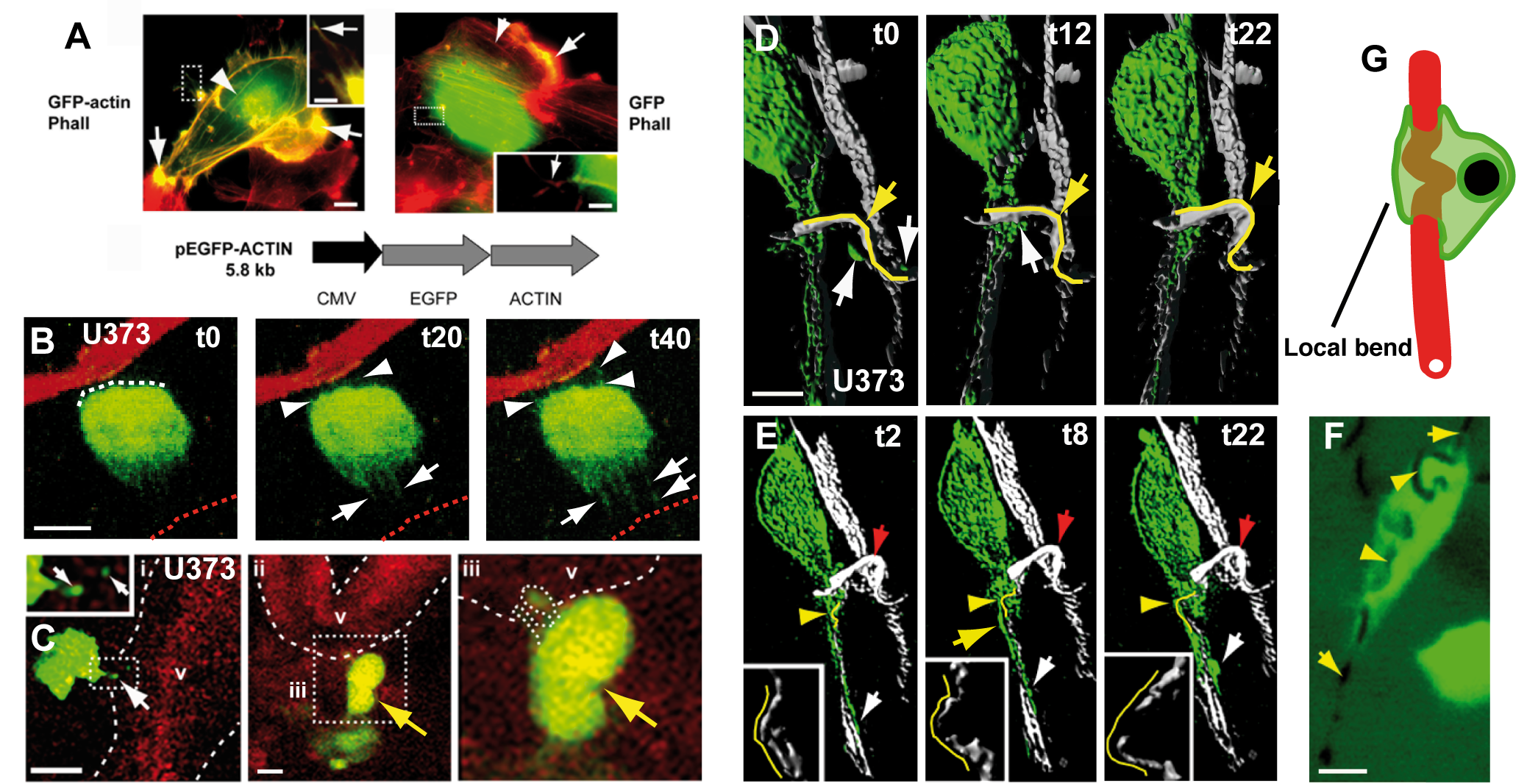

Supplement: Figure S1 — Vessel co-option and remodeling by GBM cells in brain slices. GFP-actin transfection of GBM cells allowed to investigate the actin cytoskeleton dynamics during tumor cell/vessel interaction. A, In vitro, phalloidin staining (red) of glioblastoma cells transfected with GFP-actin construct (below) shows complete overlapping (yellow) with the actin cytoskeleton (green), at intracellular level (stress fibers, white arrowhead) and in cellular protrusions, both in ruffles (white arrows) and in filopodia (arrow in inset), while GFP transfection alone shows very little co-localization in ruffles (white arrow and yellow color) and no co-localization either in stress fibers (arrowhead) or filopodia protrusions (arrow in inset). B, 2-photon video frames showing a co-opting glioblastoma cell making initial contact with a vessel (DiI-red) through cell polarization and emission of actin-enriched extensions (arrowheads); the white dotted line indicates the absence of protrusions at t0. Longer extensions with discontinuous actin (arrows) are polarized towards another vessel (red dotted line). C, Cell co-option of mouse brain meningeal vessels, following intracranial injection of GFP-actin labeled-GBM cell suspensions. Intravital imaging of the superficial neocortex confirms that injected U373 tumor cells (also labeled with CMTMR, red), after initial polarization towards blood vessels (v, DiI, red, dashed lines), emit actin-enriched thin cellular extensions (white arrow in i), which contact the vessel abluminal surface (inset: beaded organization of actin in the protrusion, arrows). Although thicker protrusions are detectable (yellow arrows in ii and iii), they always bear thinner terminal elongations that contact the vessel (dotted lines in magnification, iii). D–E, frames from two 4D rendered-confocal videos (in E only the vessel is rendered), showing U373 cells modifying blood vessels (Ink-filled, grey) in brain slices. D, an additional example of a flectopodia-linked vessel modi [file pone.0101402.s001.tif]

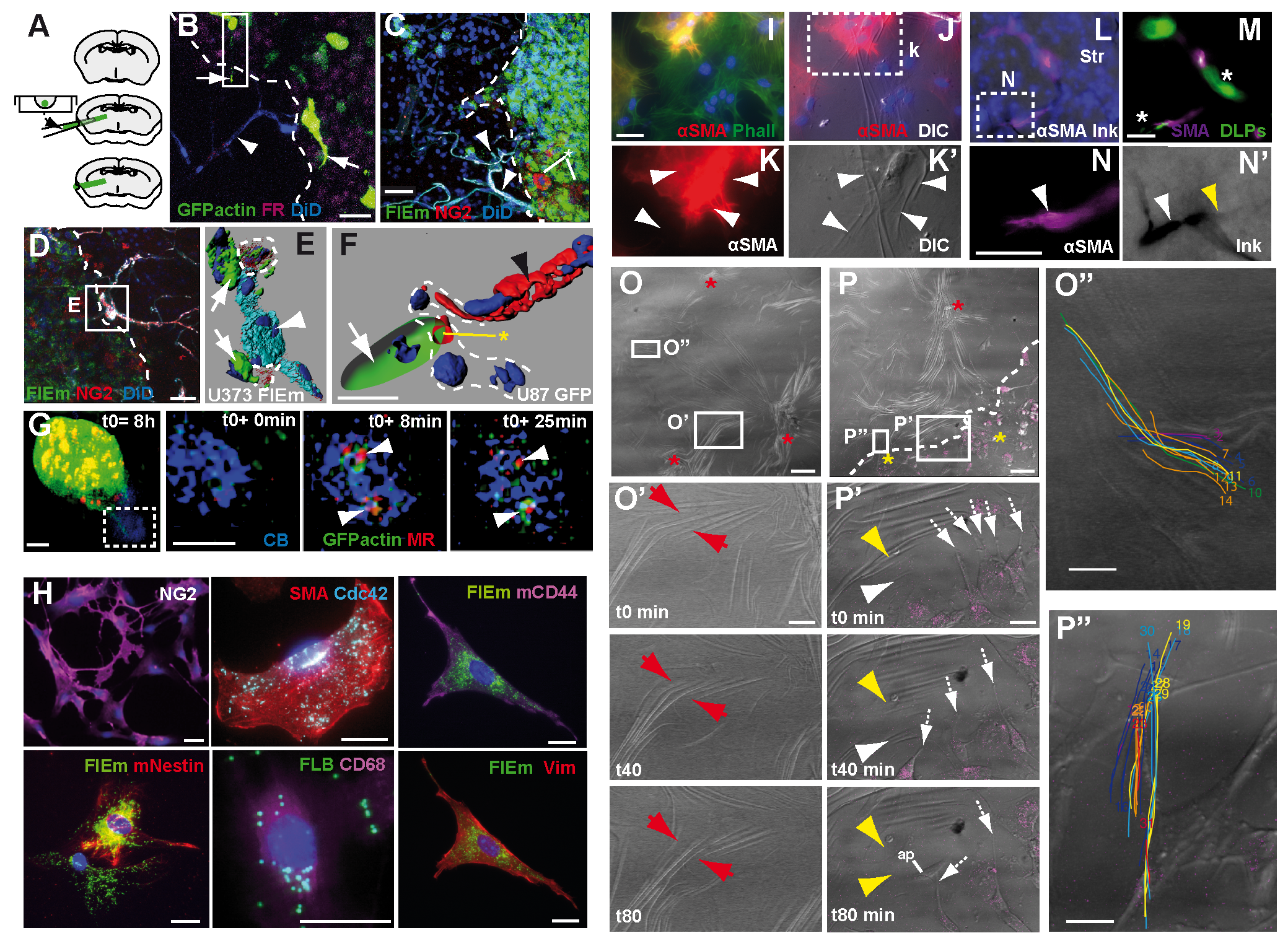

Supplement: Figure S2 — GBM cells specifically target brain pericytes in vivo and alter their contractility in vitro . A, Experimental scheme showing insertion of GBM cell-pellets into the striatum of NG2DsRedBAC-brain slices. 3D reconstructions from both a confocal video (B) and fixed samples (C–D), illustrating U373 cells (labeled either with FR-dextran/GFP-actin or FlEm Dextran) during (B) and after (C–D), respectively, the co-option of vessels (arrowheads, DiD-blue; already highly modified in C and D) at the graft/host border (dashed lines). White arrows point to GFP-actin-labeled flectopodia. Asterisk in C indicates red NG2+-cells recruited into the graft. Boxed area in B is magnified in Fig. 2A. E, 3D rendering of the boxed region in D, showing an incipient glomeruloid blood vessel (arrowhead), resulting from the interaction of GBM cells (green, white arrows) with NG2+-perivascular cells (red, bounded by dashed lines). Cell nuclei in blue (Hoechst, C–F). F, 3D rendering showing another example of a GFP-labeled GBM cell (green, white arrow) in contact with a NG2-labelled pericyte (black arrowhead) on a bent vessel segment (outlined by dashed lines), in brain slices. Yellow asterisk illustrates partial sharing of cytoplasm. G, Confocal section-video frames of a MiRu+, GFP-actin+-U373 cell seeded 8 hour earlier, showing the co-transfer (white arrowheads in magnifications of boxed area) of GFP-actin (green) and MiRu-Dextran (red) into a DLP (Cascade Blue, CB, blue). Note the similarity with the double labeled cytoplasm in the dashed areas in E. H, Purified brain pericytes grown in vitro were analyzed by immunocytochemistry for the markers indicated (in some cases were pre-labeled with FlEm-Dextran, green, or after challenge with 1 µm-fluorescent latex beads (FLB) to test for phagocytic uptake). I–K’, Heterogeneous distribution of actin proteins (phalloidin, green, in i and αSMA, red, in I–J) in pericytes plated on silicone plus human laminin. Wrinkles in magnified box (arrowheads, K’) ar [file pone.0101402.s002.tif]

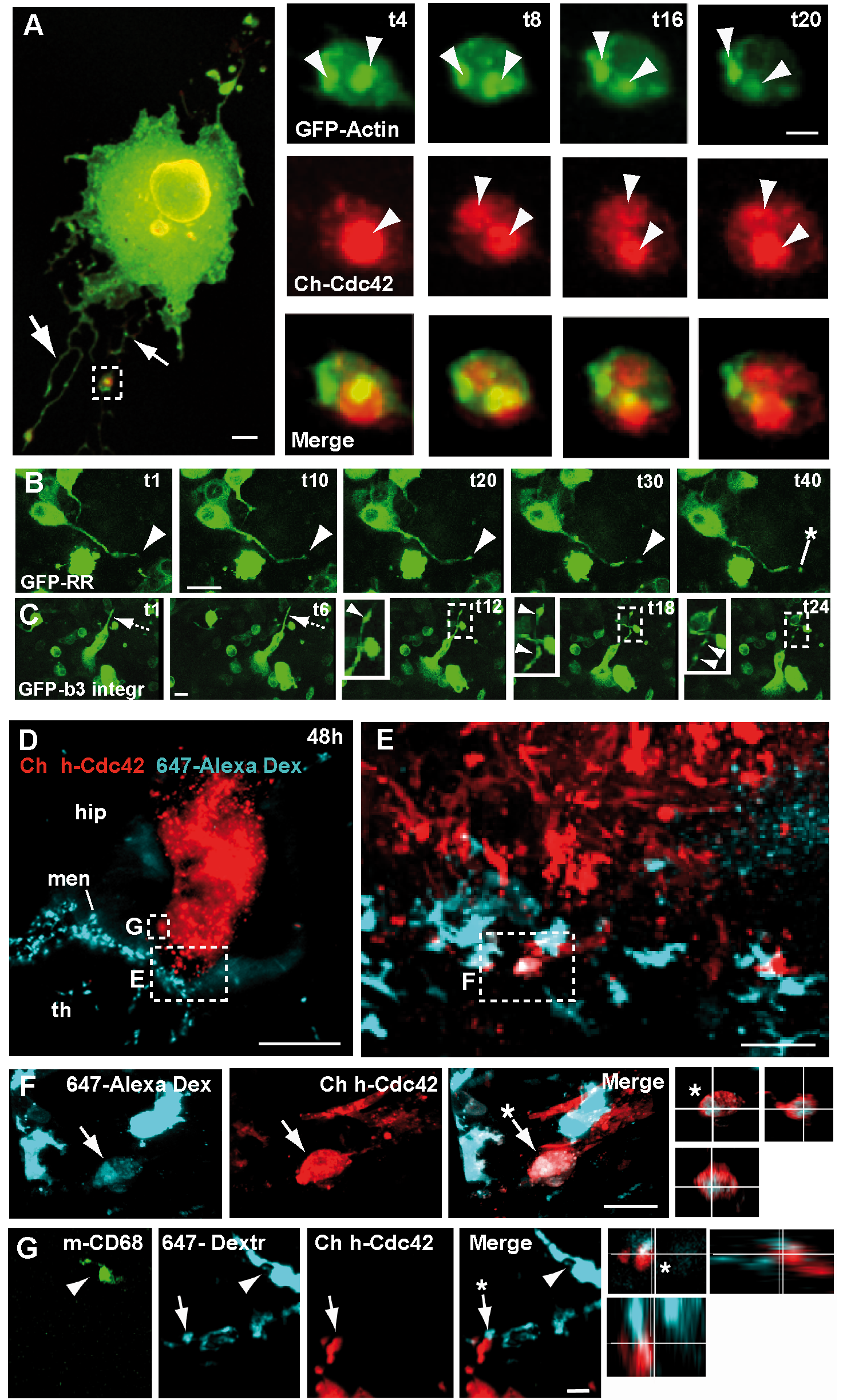

Supplement: Figure S3 — Cdc42 protein localizes in flectopodia varicosities and is transferred into pericytes in xenografts. A, Confocal video-frames of a U87 GBM cell in vitro. Co-transfection with GFPactin (green) and CherryCdc42 (Ch-Cdc42, red) reveals the striking dynamic association between actin beads and local accumulations of Cdc42 (white arrowheads in magnifications) in the cytoplasmic varicosities within the long cellular extensions (white arrows in A). Arrowheads (B) and dashed-arrows (C) indicate varicosities positive for GFP-tagged outer mitochondrial membrane protein (GFP-RR) and β3-integrin (arrowheads in insets), respectively, in the extensions of GBM cells cultured as 3D aggregates on laminin-coated glass (confocal video frames). Asterisk points to a labeled fragment released from the tip of the extension. Time in minutes. D, 3D confocal reconstruction (100 µm section) of a 2-day Ch-Cdc42-U373 cell xenograft, immunostained for the Cherry-tag (red); dextran-labeled-host cells, cyan. th, thalamus; hip, hippocampus; men, meninges. E, Graft/host interaction-zone from an adjacent section to the boxed-area in D, magnified in F; white arrows point to a cell double-labeled for Ch-Cdc42 and host-dextran. G shows localized tumour-Cdc42 transfer into a dextran-labeled, CD68–host cell (white arrows), indicating that cell/cell transfer can occur by a mechanism other than phagocytosis. White arrowheads point to a CD68+-dextran labeled-phagocyte. G is an adjacent section in the region boxed in D. Co-localizations (in F and G) are confirmed in confocal xyz-sections (white asterisks). Scale bars: 8 and 2 µm (A and magnification), 25 µm (B), 15 µm (C), 550 µm (D), 50 µm (E), 20 µm (F), 10 µm (G). (TIF) [file pone.0101402.s003.tif]

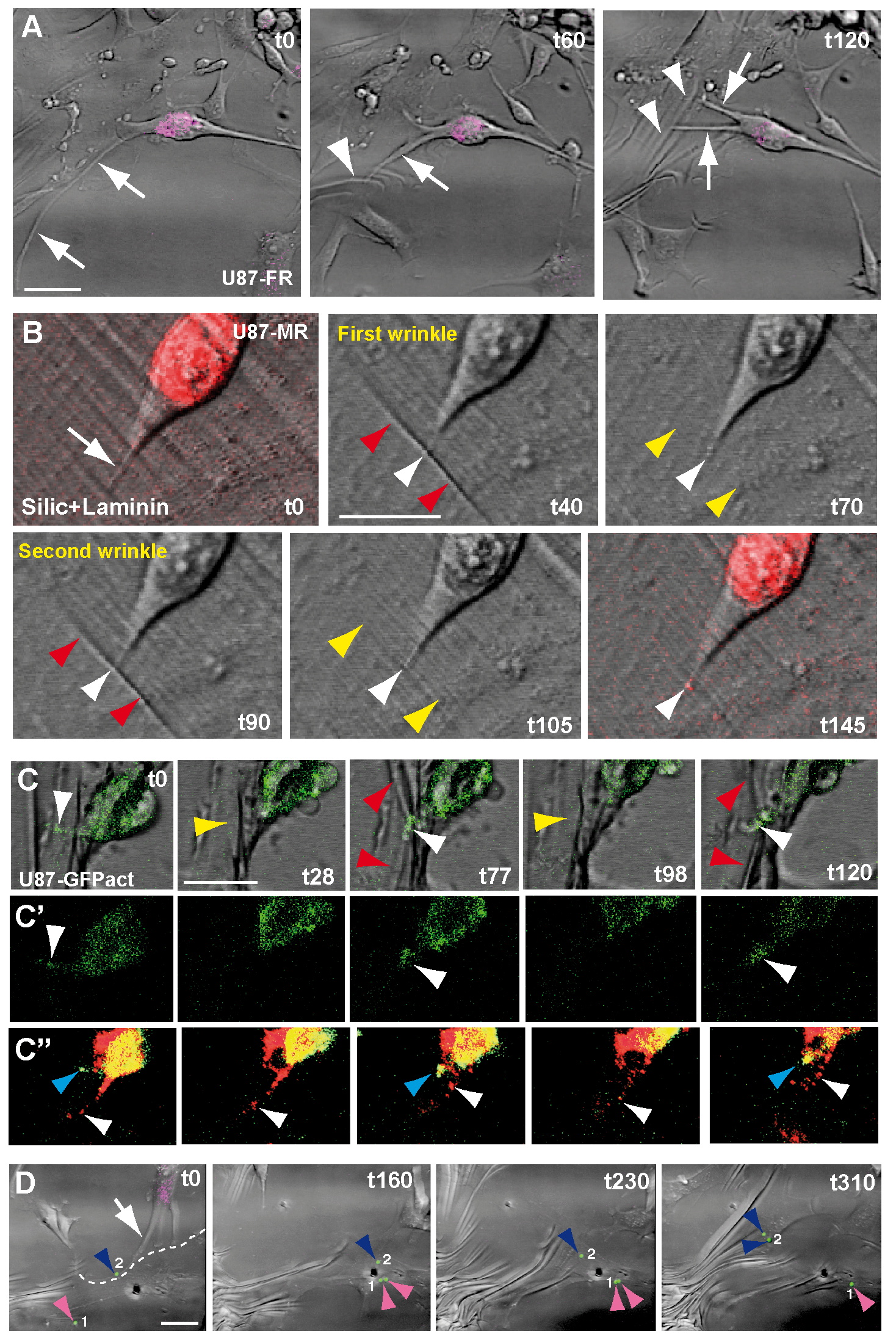

Supplement: Figure S4 — GBM cell-flectopodia modify local wrinkling patterns of pericytes plated on flexible substrates. Videos frames (combined DIC and fluorescence confocal microscopy) of GBM cell/pericyte co-cultures on laminin-coated silicone substrates. A, Flectopodia extending from a FarRed-labeled U87 cell (arrows) coincide with the presence of new wrinkles (arrowheads). B, Wrinkles, centered around terminal varicosities (white arrowheads) of the flectopodium (white arrow) extending from a MiRu-labeled-GBM cell (red color shown in first and final frames, only), are induced with a period of approximately 40–50 min (red arrowheads: presence, yellow arrowheads: absence). C, Flectopodia of two MiRu+-GBM cells (arrowheads in C” [lower magnification of C–C’]), one of which transfected with GFP-actin (green, white arrowheads in C and C’; light blue arrowheads in C”), show local accumulations of GFP-actin and dextran. Note that the waves of altered contraction of the substrate (red/yellow arrowheads in C) are in phase with periodic enrichment of actin at the tip of the flectopodium (white arrowheads in C–C’; blue arrowheads in C”). D, Cytoplasmic particles (1 and 2, indicated by green dots), either pre-existing (magenta arrowheads) or released as varicosities (dark blue arrowheads) from the tips of flectopodia (arrow) translocate and divide within the target pericyte. Time in minutes. Scale bars: 40 µm (A, D), 20 µm (B), 25 µm (C). (TIF) [file pone.0101402.s004.tif]

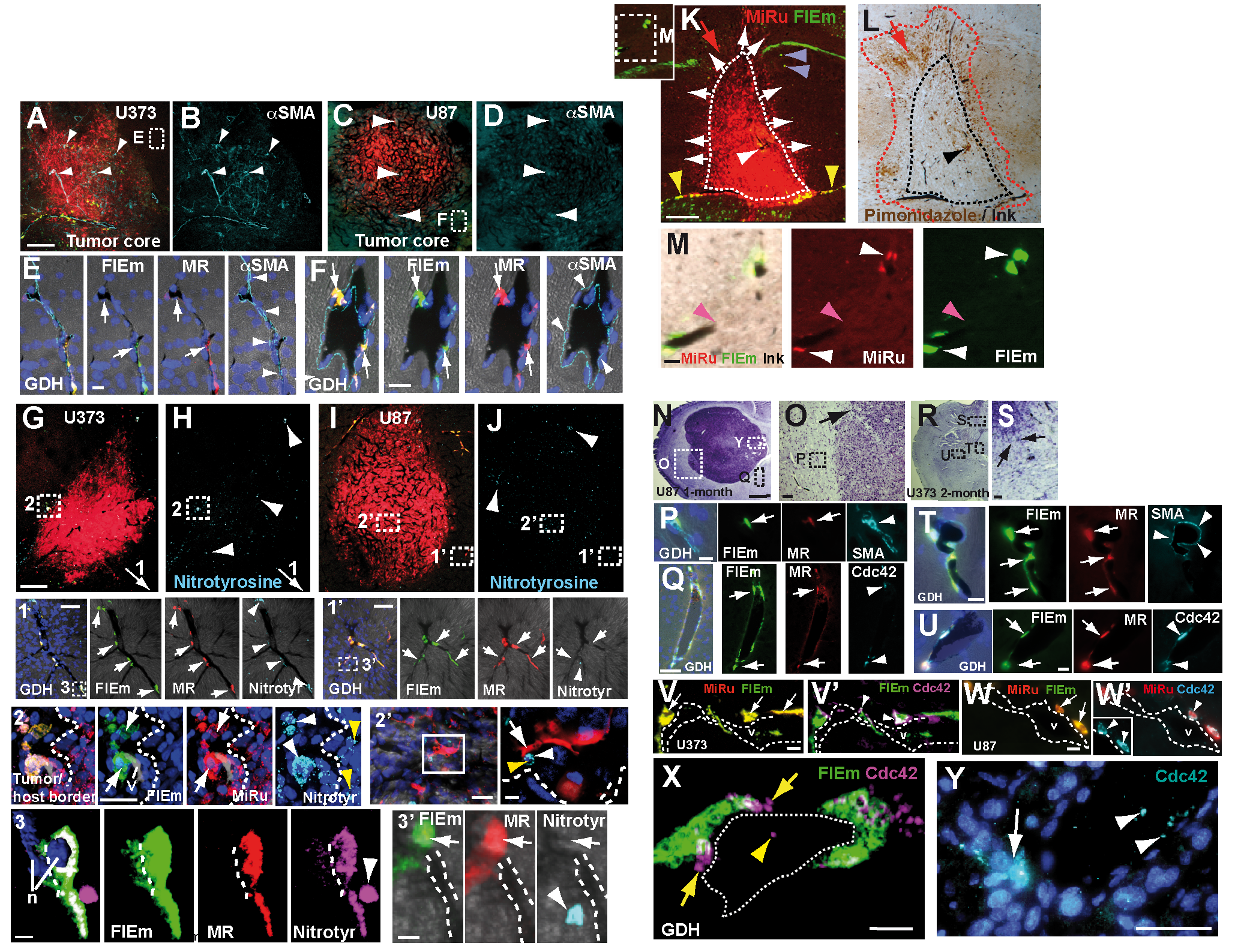

Supplement: Figure S6 — Markers of altered pericyte contractility in short and long-term GBM mouse xenografts and induced hypoxia. A and C Show triple-labeled confocal reconstructions of 120 µm-coronal sections from 7-day MiRu-labeled (red) U373 (A) and U87 (C) tumors, grafted into mice with pre-labeled DLPs (FlEm, green) and stained for αSMA (cyan). Although αSMA, a marker of contractile pericytes, is generally more prevalent in perivascular cells in U373 than U87 grafts (white arrowheads, A–D), both cell lines (E and F, boxed areas in A and C) express very high levels of αSMA (arrowheads in E–F) in GDH fusion-like hybrids (arrows), located outside the graft on highly modified vessels (Ink filled, black). Similarly, Nitrotyrosine, a marker of nitrative stress, is also present in the grafts (arrowheads in H and J), but shows its highest level (arrowheads in 1–1′) in association with αSMA+-GDH cells (arrows), accumulating either within the GDH cell itself (G1, 3) or in immediately adjacent pericytes (I1’, 3′). This differential localization of Nitrotyrosine between U373 and U87 is also evident in the tumor core, where it is found either in putative fusogenic zones at the tumor/host interface (G2) or in contact with MiRu+-cells in perivascular locations in the graft core (I2’), respectively. Infiltrating tumor cell-tongues (K, MiRu, red, white arrows) and Pimo-stained hypoxic regions (L, brown, Pimonidazole) in a 7-day U373-xenograft. White and black dotted lines: core of the implant; red dotted line: hypoxic penumbra in L. GDH cells are indicated by light blue arrowheads in K and by white arrowheads in magnifications of the inset (M). Modified vessels in M (magenta arrowheads), associated to GDH cells, connect directly to the most intense hypoxic zone (red arrows in K and L). White and black arrowheads (K and L) show a ring of Pimo staining, surrounding degenerating material in the tumor core. Yellow arrowheads in K point to type-2 cells on meninges. A continuing role for GDH cells in regul [file pone.0101402.s006.tif]

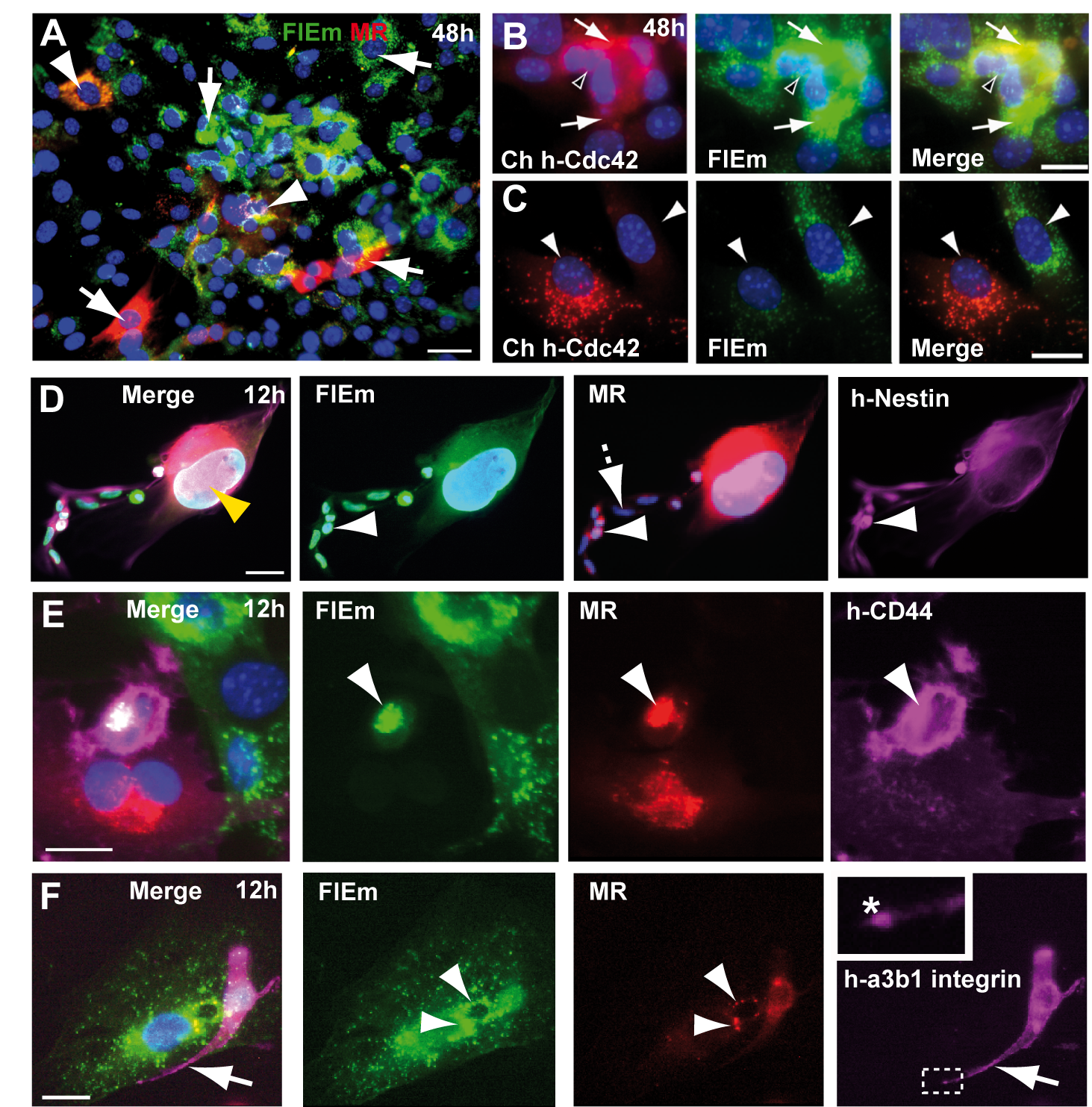

Supplement: Figure S7 — Generation of pericyte/GBM cell fusion-like hybrids in vitro . Fluorescence micrographs showing the presence of double labeled-progeny (arrowheads in A and C), 48 hours after co-culturing FlEm (green)-pericytes with GBM cells, labeled either with MiRu (MR, red, A) or Cherry-tagged Cdc42 protein (red, B–C). Arrows indicate in A parental-type single labeled-cells and in B plurinucleated (black-filled white arrowheads), putative fusogenic zones. Immunocytochemistry on double-labeled derivatives shows that two human GBM markers associated with stemness (h-Nestin and h-CD44, D and E respectively) are maintained in early stages (12 h after replating, white arrowheads). Particularly striking is the range of double-labeled cell morphologies seen at 12 h, which includes cells containing both an enormous macronucleus (yellow arrowhead in D) and multiple micronuclei (dashed arrow), each surrounded by local accumulations of co-labeled cytoplasm, suggesting both abnormal DNA replication and possible segregation of aneuploidy mini-cells. Furthermore, the differential distribution of human stem markers in the fusion-like derivatives suggests that a highly asymmetric process of cytoplasmic segregation occurs rapidly following mixing (E). In addition to complete cytoplasmic co-labeling, we also found cell/cell pairings (arrowheads in F point to double-labeled zones) that appear to involve flectopodia-like extensions (arrows). Asterisk: accumulation of α3β1 integrin at the tip of the flectopodium. Nuclei, Hoechst, blue. Scale bars: 30 µm (A), 15 µm (B, C, E), 40 µm (D), 20 µm (F). (TIF) [file pone.0101402.s007.tif]

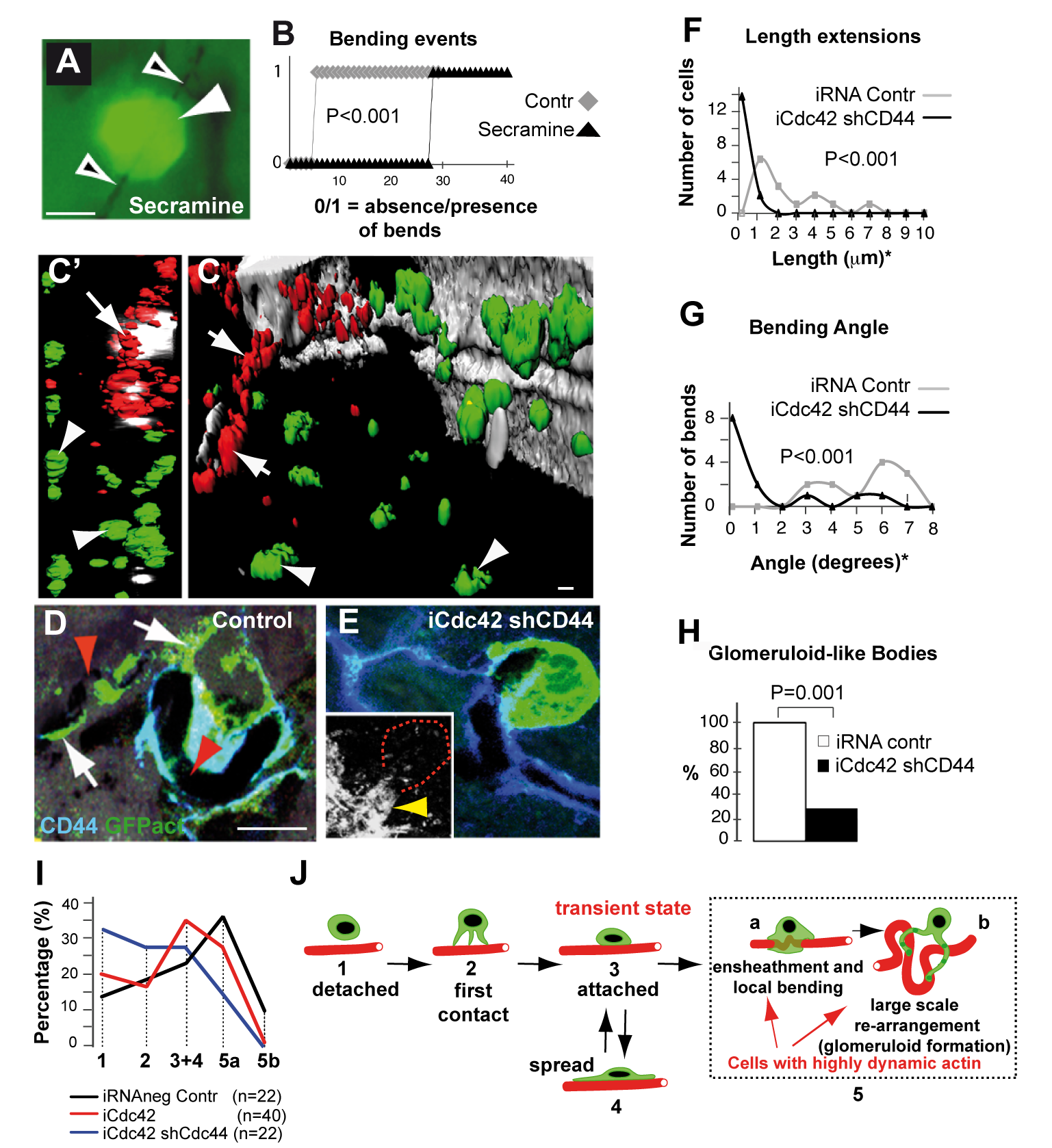

Supplement: Figure S8 — CD44 synergizes with Cdc42 during GBM cell co-option and modification of blood vessels in brain slices. A, GFP-actin transfected tumor cells (green) treated with the Cdc42-inhibitor Secramine-A appear morphologically round and not polarized (white arrowhead), on a straight vessel (black-filled white arrowheads). B, The graph shows the effect on vessel modification due to the inhibition of Cdc42 activity by Secramine-A. Controls, n = 29; Secramine, n = 42. C, Front and side views (C and C’, respectively) from the 4D rendering of a confocal video, showing a wild type-U373 cell implant (MiRu, red) next to an iCdc42 (FlEm, green)-treated implant. Wild-type cells (arrows) exit the graft on blood vessels (grey), while iCdc42-cells (arrowheads) are found scattered in the parenchyma. D, High level of CD44 (blue) at contact sites with bent vessel segments (red arrowheads) in GFP-actin-transfected GBM cells (white arrows). Double inhibition for CD44 and Cdc42 leads to a round cell phenotype (GFP-actin+, green in E; CD44 in blue). Inset shows lack (red dotted-line) of CD44-labelling (grey color), with no evidence for vessel bending (yellow arrowhead). F–H, Statistical analysis of the outcome of Cdc42 interference, coupled to down-regulation of CD44, shows stronger inhibitory effect compared to Cdc42 inhibition alone; F, controls and iCdc42 shCD44, n = 15; G, controls and inhibited, n = 13; H, controls, n = 36; treated: n = 31 (asterisks: see Methods for length/angle grouping). I–J, Quantitative effects of Cdc42 and CD44 interference (graph, left hand side) on the frequency of 5 tumor cell morphologies in brain slices (hypothetical temporal sequence, right hand side). Scale bars: 10 µm (A, C–D). (TIF) [file pone.0101402.s008.tif]

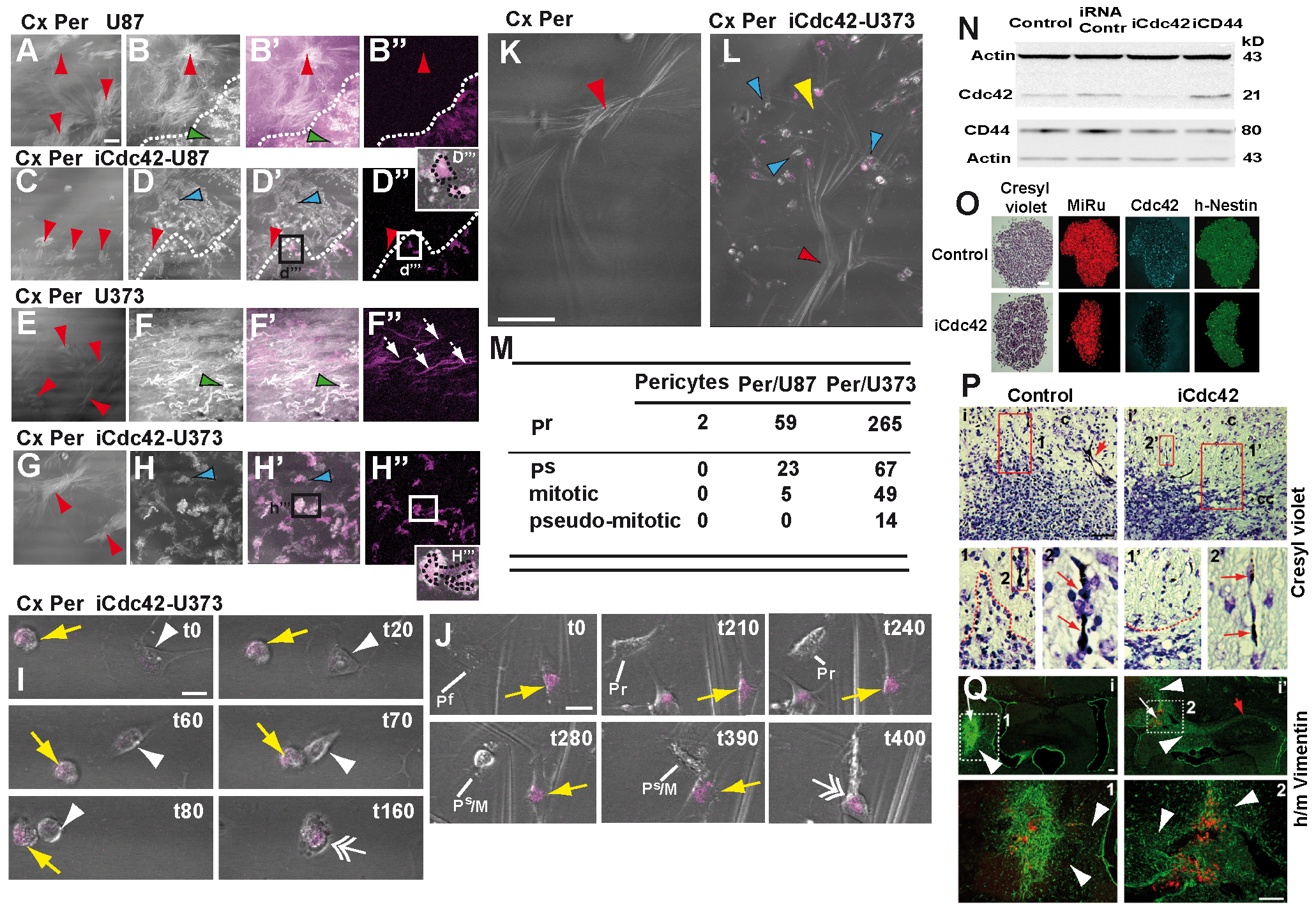

Supplement: Figure S9 — Inhibition of Cdc42 in GBM cells converts contractile pericytes into a phagocytic, macrophage-like phenotype on silicone substrates and leads to tumor suppression in vivo . A–H”, To provide a sketch of the global dynamics of GBM cell/pericyte interactions, we created a single 2D image (maximum projection in both z and time axes) for each 4D movie of pericytes alone (A, C, E, and G; DIC optics) and co-cultures, using FarRed-labeled (FR)-GBM cells, either U87 (B–B”), iCdc42-U87 (D–D”), U373 (F–F”) and iCdc42-U373 (H–H”) (X, X’ and X” represent DIC, DIC plus fluorescence and fluorescence alone, respectively). This analysis showed a striking difference between the behavior of the two cell lines. U87 cells occupy a contained region of the field, with a clearly discernable boundary (dotted line in B”), reflecting a random cell movement within a circumscribed area. In contrast, U373 cells produce more or less parallel lines distributed throughout the field (arrows in F”), which represent the trajectories of directed cell migration. Red arrowheads: node regions present in the field, before and after GBM cell addition. Wild-type GBM cells also cause the activation of flat and contractile pericytes (Pf) into raised (Pr) and spheroidal pericytes (Ps, green filled-black arrowheads in B–B’ and F–F’), normally characteristic of plastic anti-node regions of pericytes plated alone (tabulated in M). Ps pericytes also include mitotic and pseudo-mitotic cells (those with a transient cleavage furrow). The analysis of 4D-2D projection patterns for GBM cells inhibited for Cdc42 showed that the overall field features for both U373 and U87 cells were completely transformed. First, the normal organization of U87 as a circumscribed block and of U373 in quasi-parallel lines is replaced, in each case, by discrete compact FR-cell patches (D” and H”, respectively), reflecting locally confined, rotating trajectories. Second, we found an overall reduction in wrinkling activity, correlated with a f [file pone.0101402.s009.tif]

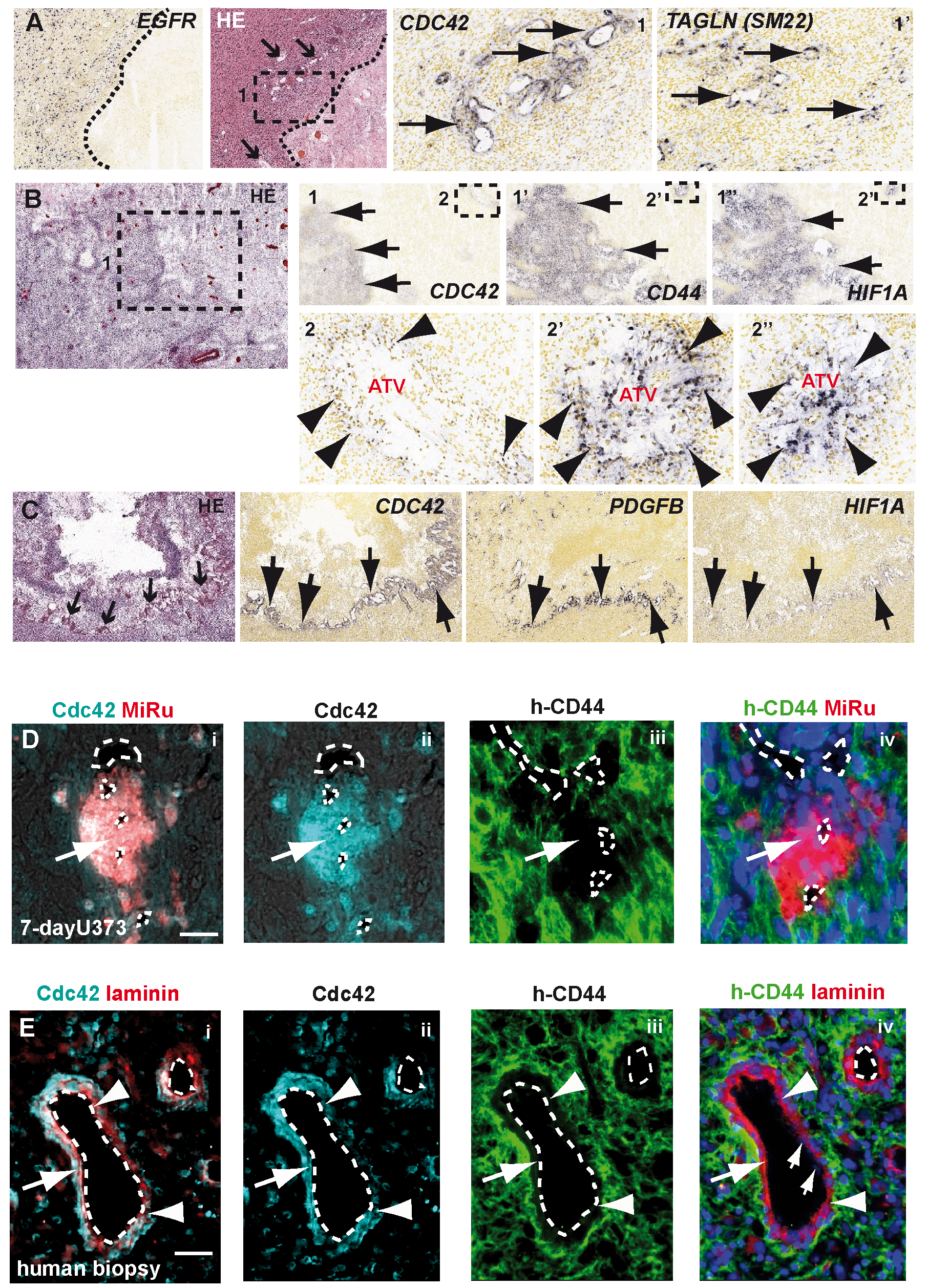

Supplement: Figure S10 — A synexpression group of perivascular contractile/hypoxia cell markers in human glioma. A–C, Three independent glioblastoma tumors, taken from the Allen Brain Atlas database (www.brain-map.org). Note co-expression of the markers indicated (arrows and arrowheads in magnifications) in abnormal tumor vasculature (small arrows in Haematoxylin and Eosin stainings, HE), located at the border between the least (strongly stained) and more (weakly stained) differentiated tumor tissue (shown as dotted lines in A). The comparison of a presumptive fusion zone in the core of a 7-day U373-xenograft (MiRu+, D) with an anomalous vascular structure in a human GBM biopsy (E) shows the similarity of marker expression around highly modified vessels, raising the interesting possibility that the hypertrophic Cdc42+, laminin-rich-perivascular regions are the sites of ongoing GBM cell/pericyte amalgamation (white arrows in D; in E white arrows point to endothelium-free, hypertrophic perivascular layer). Dotted lines indicate the lumen of abnormal vessels; endothelial cell nuclei (E-iv, small white arrows) are visible only in that part of the vessel-wall lacking Cdc42 (white arrowheads). Nuclei in blue, Hoechst. Scale bars: 17 µm (D), 40 µm (E). (TIF) [file pone.0101402.s010.tif]
