# Supplementary material for: Glioblastoma: A Pathogenic Crosstalk between Tumor Cells and Pericytes
Source: PLoS One. 2014 Jul 17;9(7):e101402. doi: 10.1371/journal.pone.0101402 (PMC4102477; doi:10.1371/journal.pone.0101402)
Supplement: Discussion S1 — Tumor cell/pericyte fusion. (DOCX) [file pone.0101402.s021.docx]

**Supporting Discussion**

**Tumor cell/pericyte fusion.**

A surprising result of our work is the discovery of fusion-like cells formed between tumor cells and pericytes, both during the reorganization of xenografts and in co-culture experiments. Brain pericytes have an additional role as macrophage-like cells. In cancer, the macrophage is generally considered to be the tumor cell fusion-partner [71]. Macrophages are themselves fusogenic and can undergo homotypic fusion [72] to generate multinucleated macrophage-giant cells and bone re-absorbing osteoclasts [73]. Macrophages are also phagocytic and we found that DLPs can express variable amounts of the macrophage and phagocytic marker CD68 (data not shown). Therefore, while it seems likely that the host cell-type from which ‘Glioblastoma DLP Hybrid’ (GDH) cells derive are macrophage-like pericytes, it is unclear if GDH cells are the result of a fusion or a phagocytic event, bearing in mind that fusogenic macrophages maintain high CD68 protein expression [74].

Cell fusion is thought to be a route to generate cancer cell diversity during tumor progression [75], [76], [71]. Fused tetraploid cells often undergo multipolar division and are eliminated at a p53-dependent checkpoint, because of their chromosomal instability [77], [78]. Since U373 and U87 cells both lack p53 function, due either to the mutation of TP53 itself (U373) or of the TP53-regulator CDKN2A (p16Ink4A) (U87) [79], [80], the tetraploid checkpoint in fusion hybrids could be compromised. The possibility, then, exists for novel GBM cell/pericyte aneuploid fusion derivatives to become established and proliferate. We identified double-labeled cells in the core of 7-day xenografts (type-1f). Moreover, our data strongly suggest the occurrence of tumor cell/pericyte fusion, including the presence of pluri-nucleated derivatives, in *in vitro* co-cultures and on laminin-coated silicone substrates. Based on our work, the interaction of wild-type GBM cells with pericytes during blood vessel co-option leads to cytoplasmic mixing, both through local transfer and wholesale fusion.

These two events could generate novel tumor cell variants, assuming that nuclear and cytoplasmic determinants segregate independently and that cytoplasmic determinants have a dominant effect on cell behavior. In our hypothetical scheme (Figure S11 E-F), TP1, TP2, Hy2 and Hy3 carry different combinations of nuclear and cytoplasmic tumor traits from the parental cell types and may contribute to cellular diversification during tumor progression. Accordingly, fusion should also produce the parental type-derivatives Hy1 and Hy4. Tumor propagating cells, specified by the inheritance of tumor cytoplasmic determinants, are represented by one parental tumor-type (Hy4) and two novel recombinants (TP2 and Hy2), both of which have lost the tumor nucleus. These latter two types could correspond to the recruited, tumor propagating cells described by Fomchenko et al. [38]. Pericyte-like cells, specified by the inheritance of pericyte cytoplasmic determinants, are represented by one parental pericyte-type (Hy1) and two novel recombinants (TP1 and Hy3), both of which have inherited the tumor nucleus. TP1 and Hy3 may be analogous to the tumor trans-differentiated perivascular cells described by Cheng et al. [23]. Although it is difficult to make a precise correlation, the work presented here suggests a possible identification of the hypothesized segregated, fusion hybrids and those found in our xenografts. Basing on the cellular features described above, it is plausible that the tumor parental-like cell types Hy1 and Hy4 correspond to our type-2 and type-1f cells, while the recombinant cell types TP1 and Hy2 are consistent with our type-1 and GDH cells, respectively. In addition and central to the co-option process, TP2 may represent host pericytes with altered contractility, which have inherited tumor cell cytoplasm.

**References for Supporting Discussion**

71. Dittmar T, Nagler C, Niggermann B, Zänker KS (2013) The dark side of stem cells: triggering cancer progression by cell fusion. Curr Mol Med 13: 735-750.

72. Miyamoto T (2013) STATs and macrophage fusion. JAKSTAT 2:e24777. doi: 10.4161/jkst.24777.

73. Miyamoto T (2011) Regulators of osteoclast differentiation and cell-cell fusion. Keio J Med 60: 101-105.

74. McNally AK, Anderson JM (2011) Foreign body-type multinucleated giant cells induced by interleukin-4 express select lymphocyte co-stimulatory molecules and are phenotypically distinct from osteoclasts and dendritic cells. Exp Mol Pathol 91: 673-681.

75. Pawelek JM, Chakraborty AK (2008) Fusion of tumour cells with bone marrow-derived cells: a unifying explanation for metastasis. Nat Rev Cancer 8: 377-386.

76. Lu X, Kang Y (2009) Cell fusion as a hidden force in tumor progression. Cancer Res 69: 8536-8539.

77. Vitale I, Senovilla L, Jemaà M, Michaud M, Galluzzi L, et al. (2010) Multipolar mitosis of tetraploid cells: inhibition by p53 and dependency on Mos. EMBO J 29: 1272-1284.

78. Ho CC, Hau PM, Marxer M, Poon RY (2010) The requirement of p53 for maintaining chromosomal stability during tetraploidization. Oncotarget 1: 583-595.

79. Clark MJ, Homer N, O’Connor BD, Chen Z, Eskin A, et al. (2010) U87MG decoded: the genomic sequence of a cytogenetically aberrant human cancer cell line. PLoS Genet 6:e1000832. doi: 10.1371*/*journal.pgen.1000832.

80. Shono T, Tofilon PJ, Schaefer TS, Parikh D, Liu TJ, et al. (2002) Apoptosis induced by adenovirus-mediated p53 gene transfer in human glioma correlates with site-specific phosphorylation. Cancer Res 62: 1069-1076.
